# Assigning function to active site residues of *Schistosoma mansoni* thioredoxin/glutathione reductase from analysis of transient state reductive half-reactions with variant forms of the enzyme

**DOI:** 10.3389/fmolb.2023.1258333

**Published:** 2023-09-13

**Authors:** Madison M. Smith, Graham R. Moran

**Affiliations:** Department of Chemistry and Biochemistry, Loyola University Chicago, Chicago, IL, United States

**Keywords:** flavoprotein, disulfide, reductase, transient state, kinetics, variant

## Abstract

Thioredoxin/glutathione reductase (TGR) from the platyhelminthic parasitic worms has recently been identified as a drug target for the treatment of schistosomiasis. Schistosomes lack catalase, and so are heavily reliant on the regeneration of reduced thioredoxin (Trx) and glutathione (GSH) to reduce peroxiredoxins that ameliorate oxidative damage from hydrogen peroxide generated by the host immune response. This study focuses on the characterization of the catalytic mechanism of *Schistosoma mansoni* TGR (SmTGR). Variant forms of SmTGR were studied to assign the function of residues that participate in the electron distribution chain within the enzyme. Using anaerobic transient state spectrophotometric methods, redox changes for the FAD and NADPH were observed and the function of specific residues was defined from observation of charge transfer absorption transitions that are indicative of specific complexations and redox states. The C159S variant prevented distribution of electrons beyond the flavin and as such did not accumulate thiolate-FAD charge transfer absorption. The lack of this absorption facilitated observation of a new charge transfer absorption consistent with proximity of NADPH and FAD. The C159S variant was used to confine electrons from NADPH at the flavin, and it was shown that NADPH and FAD exchange hydride in both directions and come to an equilibrium that yields only fractional FAD reduction, suggesting that both have similar reduction potentials. Mutation of U597 to serine resulted in sustained thiolate-FAD charge transfer absorption and loss of the ability to reduce Trx, indicating that the C596-U597 disulfide functions in the catalytic sequence to receive electrons from the C154 C159 pair and distribute them to Trx. No kinetic evidence for a loss or change in function associated with the distal C28-C31 disulfide was observed when the C31S variant reductive half-reaction was observed. The Y296A variant was shown to slow the rate of but increase extent of reduction of the flavin, and the dissociation of NADP^+^. The H571 residue was confirmed to be the residue responsible for the deprotonation of the C159 thiol, increasing its reactivity and generating the prominent thiolate-FAD charge transfer absorption that accumulates with oxidation of the flavin.

## Introduction

Human schistosomiasis is a prevalent parasitic disease that afflicts roughly 240 million people worldwide and is caused by trematode worms of the genus *Schistosoma* (Skelly, 2008; Colley et al., 2014; WHO, 2023). The disease tends to impact poorer communities in mostly tropical and subtropical regions (van der Werf et al., 2003a; van der Werf et al., 2003b; Kokaliaris et al., 2022). Initial schistosomiasis infection occurs after exposure to water sources that contain schistosome larvae (cercariae), which readily penetrate human skin to infiltrate a host (Steinmann et al., 2006). Once within the host, the immature flukes will mature into adult worms ultimately producing eggs which migrate throughout the body becoming imbedded in various organs (Skelly, 2008; Colley et al., 2014). Transmission between individuals occurs when worm eggs enter the intestinal tract or bladder and are eliminated back into the environment. Praziquantel is a safe anthelmintic drug developed in the 1970s (Jeziorski and Greenberg, 2006; Doenhoff et al., 2008; Wang et al., 2012; Kokaliaris et al., 2022). This drug activates a transient receptor potential ion channel (TRPM) in schistosomes and the resulting movement of calcium leads to adult worm paralysis and death (Jeziorski and Greenberg, 2006). However, within more poverty-stricken regions, where clean water is less accessible and praziquantel supply is limited, individuals often become reinfected. Untreated or recurrent schistosomiasis infection leads to organ impairment caused by extensive fibrosis as a result of the host’s own immune response to the parasite, along with bacterial superinfections, bladder carcinomas, diminishment of reproductive health, increased risk of other infectious diseases, anemia, and malnutrition that collectively result in significant morbidity (WHO, 2023). Given that praziquantel is the only effective treatment for schistosomiasis, there is an increasing concern that drug resistance may become more prevalent (Utzinger et al., 2009; Wang et al., 2012). More recently, pursuit of alternative antiparasitic drugs have been made possible by a more in-depth investigation of metabolic pathways specific to schistosomes and by direct study of TGR (Silvestri et al., 2018; Fata et al., 2021; Petukhova et al., 2023).

**SCHEME 1:**
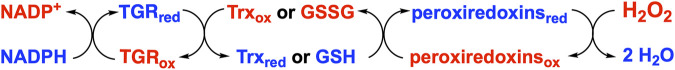
Detoxifiaction of reactive oxygen species in *Schistosoma* spp.

All organisms have mechanisms to eliminate reactive oxygen species (ROS) generated from metabolism and external oxidative stresses. However, parasitic schistosomes are highly dependent on their ROS detoxification processes as they are exposed to the host’s immune response (Colley and Secor, 2014). Paradoxically, schistosomes lack catalase and rely exclusively on reduced forms of glutathione (GSH) and thioredoxin (Trx_red_) to (re)generate reduced forms of peroxiredoxins to ameliorate hydrogen peroxide (Scheme 1) (Kuntz et al., 2007; Williams et al., 2013; Tripathi et al., 2017). In most eukaryotes, intracellular levels of GSH and Trx_red_ are maintained by two enzymes, glutathione reductase (GR) and thioredoxin reductase (TrxR). However, a single enzyme, common to *Schistosoma spp*., was identified to possess both GR and TrxR activities (Alger and Williams, 2002; Salinas et al., 2004; Bonilla et al., 2008; Bonilla et al., 2011). In addition, RNA interference has identified this enzyme, thioredoxin/glutathione reductase (TGR) (Kuntz et al., 2007), as necessary for the parasites survival and therefore is a potential drug target to kill schistosomes.

**FIGURE 1:**
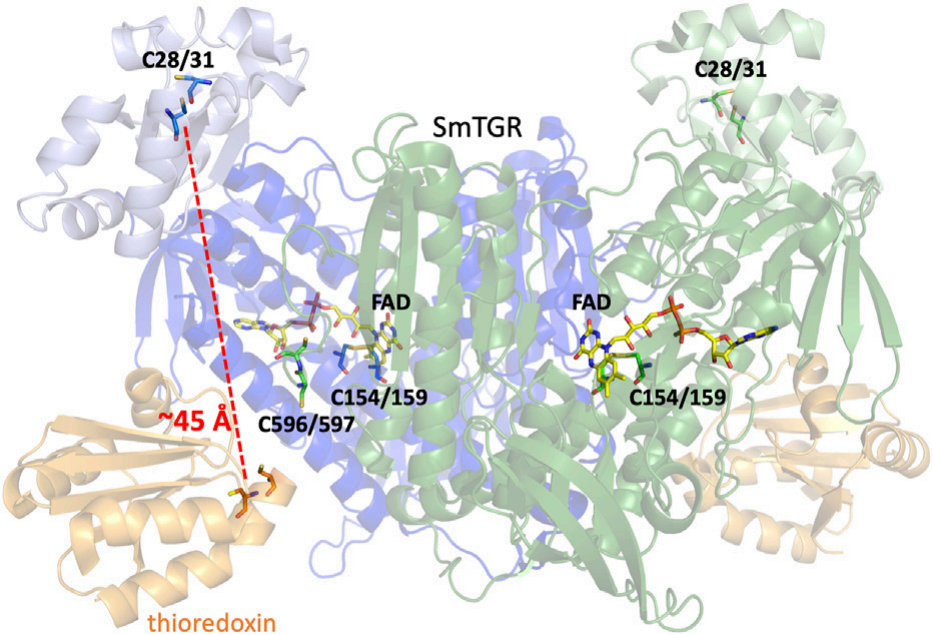
Dimeric SmTGR Crystal Structure. The SmTGR variant U597S was crystallized in the presence of NADPH, yielding the reduced form (PDB 2 × 8C). This is currently the only crystal structure with a C-terminal 596–597 disulfide resolved. Subunit 1 is displayed in blue, with the TrxR domain (dark blue) distinguished from the GR domain (light blue). Similarly, the second subunit is represented but in green. All relevant cysteine pairs and the FAD molecule are labeled. Additionally, by overlaying the structure of the plasmodium TrxR and Trx complex (PDB 4J56) provides a visual representation of the proposed Trx (orange) binding site in SmTGR.

TGR from *Schistosoma mansoni* (SmTGR) is a homodimeric, flavin-dependent enzyme which can accept electrons from nicotinamide adenine dinucleotide phosphate (NADPH) and reduce both oxidized glutathione (GSSG) and thioredoxin (Trx_ox_) (Scheme 1). Each subunit contains a non-covalently bound flavin adenine dinucleotide (FAD) and several cysteine pairs which are thought to participate in series in the displacement of electrons within the enzyme by disulfide exchange reactions (Figure 1) (Sandalova et al., 2001; Angelucci et al., 2010). The secondary structure of TGR closely resembles that of GR and TrxR, with each of these enzymes also containing an FAD and conserved redox active disulfide pairs. GR has been identified to contain one catalytic cysteine pair (Pai et al., 1988; Schulz and Karplus, 1988), TrxR to contain two (Fritz-Wolf et al., 2013), and SmTGR to contain three. Interestingly, one of the redox active pairs of SmTGR incorporates a selenocysteine residue. Although in previous studies the wild type (selenocysteine containing) enzyme is noted as being approximately 4-fold faster (Alger and Williams, 2002; Huang et al., 2011), the U597C variant has frequently been utilized for crystallographic and kinetic studies of SmTGR to allow for the use of conventional heterologous expression methods. Based on the available X-ray crystal structures and steady-state analysis of various SmTGR variants, the residues that are proposed to participate in disulfide exchange are the C154-C159, C596-C597, and C28-C31 pairs (Figure 1).

As SmTGR can act as both a TrxR and GR, when comparing the secondary structure of SmTGR with mammalian TrxR and GR one can infer which portion of the enzyme is likely responsible for which chemistry. In Figure 1, the SmTGR U597C dimer is shown, with subunit 1 in blue and subunit 2 depicted in green. The bulk of the structure closely resembles TrxR enzymes and these portions are darkly colored, with the appended GR domain of TGR being lightly colored. This TrxR core of TGR has the NADPH binding pocket, FAD, C154-C159 disulfide, and cradles the C596-C597 disulfide of the adjacent subunit of the dimer. While currently no structure of SmTGR•Trx complex exists, when aligning the available crystal structure of plasmodium TrxR•Trx (PDB 4J56) with SmTGR one can infer where Trx (shown in orange) binds to SmTGR (Figure 1). Interestingly, although mammalian GR somewhat resembles the TrxR domain of SmTGR, the GR domain of SmTGR has the fold of Trx and is appended to the N-terminus of the TrxR core of SmTGR, containing the C28-C31 pair (Angelucci et al., 2010; Huang et al., 2011) (Figure 1). Although this region of the enzyme has been confirmed to bind GSH and the C28-C31 pair has been observed to be necessary for the reduction of GSSG, there is no explicit evidence of how electrons are transmitted to the GR domain. The prevailing proposal is that electron transport between the two domains is dependent on the disordered and mobile C-terminal loop that terminates with the C596-U597 pair.

Recently, a transient-state characterization of *Sm*TGR U597C reductive half-reaction was completed (Smith et al., 2023). Utilizing the spectrophotometric properties of the FAD and charge transfer intermediate species, a more direct depiction of the electron displacement within SmTGR was demonstrated. Upon the introduction of NADPH, rapid fractional reduction of the flavin was observed, followed by several reoxidation phases. It was determined that after the flavin was reduced, electrons would quickly be transferred to reduce the C154-C159 disulfide. NADP^+^ would dissociate allowing for the deprotonation of one of the thiols generated, resulting in the formation of a broadly absorbing, thiolate-FAD charge transfer. Electrons could then be observed to shuffle further into the enzyme by monitoring the staged reoxidation of the flavin and the loss of this charge transfer absorption. As all species involved in this catalytic mechanism (NADPH, FAD, disulfide pairs, and oxidants) are redox active and it was determined that all discerned states were quasi-equilibrium states that include combinations of extents of oxidation and reduction for all redox active species.

**FIGURE 2:**
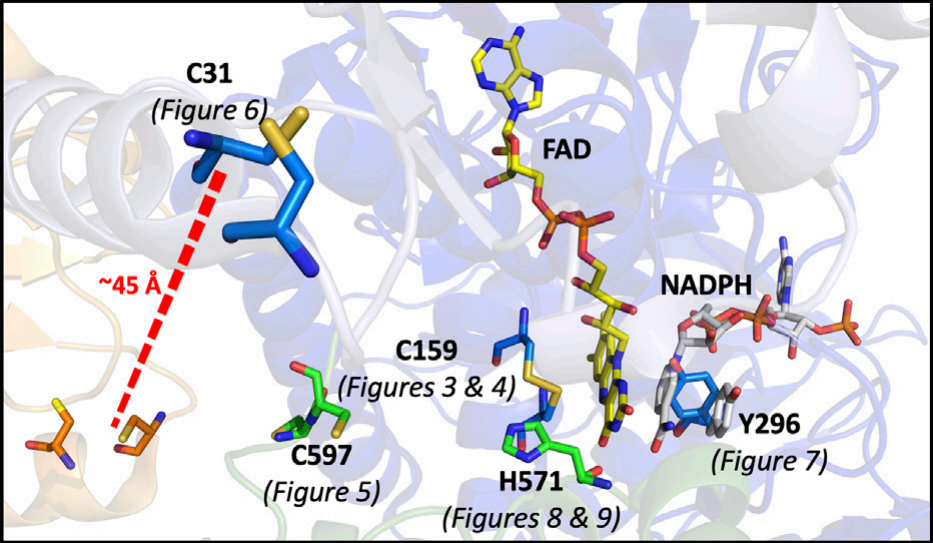
Catalytically Relevant Residues of SmTGR. The image highlights the residues that are the focus of this study. In this figure subunit A is shown in blue, subunit B is shown in green, the FAD molecule shown in yellow. The residues altered by mutation in this study are highlighted (C597 and H571 from subunit two) and the figure numbers associated with each variant are shown below. An additional crystal structure (PDB 2X99) was used to demonstrate the orientation of NADPH (grey) parallel to the FAD isoalloxazine ring and portray the open/parallel conformation of the Y296 (grey) residue when NADPH is bound.

In this study, a number of SmTGR variants were produced to develop evidence for the role of specific active site residues and thus confirm the pathway of electron transmission, while also attempting to perturb and/or reveal other elements of the catalytic mechanism (Figure 2).

These kinetic studies of SmTGR occur with the U597C variant background. This study will focus on point mutations made to render each of the proposed redox active disulfide pairs inactive; including the study of the C159S, U597S, and C31S variants. Additionally, alanine mutants of both H571 and Y296 were made. Available crystal structures suggest that due to its proximity to the C154-C159 pair, H571 acts as a base for the deprotonation that results in both heightened reactivity and the formation of a thiolate-FAD charge transfer. The Y296 residue has been demonstrated to be involved in a conformational change that occurs with NADPH binding (Silvestri et al., 2018), therefore the mutation of this residue was designed to perturb both formation of the NADPH•SmTGR complex and FAD reduction.

## Materials and methods

### Materials and quantitation

NADPH was purchased from RPI Research Products. Competent BL21 (DE3) cells were obtained from New England Biolabs. Dipotassium hydrogen phosphate (KPi) was purchased from HiMedia Laboratories. Sodium chloride (NaCl), 2-(N-morpholino)ethanesulfonic acid (MES), 2-amino-2-(hydroxymethyl)propane-1,3-diol (tris base), oxidized nicotinamide adenine dinucleotide phosphate (NADP^+^), the Miller formulation of lysogeny broth (LB) powder, sodium dodecyl sulfate (SDS), and isopropyl-β-thiogalactopyranoside (IPTG) were purchased from Fisher Scientific. Talon superflow resin was from Cytvia. Kanamycin and agar powder were acquired from Alfa Aesar. Flavin adenine dinucleotide (FAD), imidazole, acetic acid, deuterium oxide (D_2_O), and sodium dithionite were from Acros. *Aspergillus niger* catalase was purchased from Sigma-Aldrich. Phosphoric acid was purchased from VWR International.

Where possible, concentrations were determined spectrophotometrically using the following extinction coefficients: NADPH; ε_340 nm_ = 6,220 M^−1^cm^−1^, NADP^+^; ε_260 nm_ = 17,800 M^−1^cm^−1^, thioredoxin (SmTrx); ε_280 nm_ = 9,500 M^−1^cm^−1^. The extinction coefficient of each SmTGR variant was determined by methods previously reported (Smith et al., 2023): SmTGR U597C; ε_463 nm_ = 11,700 M^−1^cm^−1^, SmTGR C159S; ε_445 nm_ = 12,600 M^−1^cm^−1^, SmTGR U597S; ε_463 nm_ = 11,500 M^−1^cm^−1^, SmTGR C31S; ε_463 nm_ = 11,300 M^−1^cm^−1^, SmTGR H571A; ε_460 nm_ = 11,300 M^−1^cm^−1^, SmTGR Y296A; ε_455 nm_ = 11,500 M^−1^cm^−1^. All reactant concentrations indicated in this text are post-mixing.

### Expression and purification of SmTGR variants

The method of expressing and purifying all SmTGR variants were adapted from the previously established protocols utilized to isolate the U597C SmTGR variant (Smith et al., 2023). The variants of interest include Cys159Ser (C159S), Cys597Ser (U597S), Cys31Ser (C31S), His571Ala (H571A), and Tyr296Ala (Y296A). All variants studied also include the U597C mutation, therefore all enzymes listed here are double variants. However, for simplicity, throughout this manuscript variants will be identified only by alteration of the specific residue under study, excluding reference to the U597C mutation. The gene of interest was synthesized and subcloned into the pET28a + expression plasmid by Genscript using NdeI and XhoI restriction sites, placing the gene in phase with the sequence coding for an N-terminal 6His-tag linked by a thrombin cleavage site. The plasmid was transformed into competent BL21 (DE3) *Escherichia coli* cells. One colony was removed and added to an LB culture (containing 25 μg/mL kanamycin) and grown with shaking at 220 rpm at 37°C. Once the culture exhibited signs of turbidity, cell stocks (1 mL) were prepared by mixing 400 µL of 0.22 µm filtered 50% glycerol with 600 µL aliquots of the culture and stored in −80°C.

To express SmTGR variants, 100 µL of transformed BL21 (DE3) cells containing the corresponding plasmid were spread onto an LB agar plate (containing 25 μg/mL kanamycin selection) and incubated for 16–18 h at 37°C. Sterile LB was used to resuspend the cell lawns and this slurry was used to inoculate 1 L of LB broth (also containing 25 μg/mL kanamycin). The cells were grown with shaking (220 rpm) at 37°C to an optical density at 600 nm of 0.8. The temperature was reduced to 25°C and the media was allowed to equilibrate for 1 h, after which 100 µM IPTG was added to induce protein expression. The culture was incubated at this temperature with shaking for an additional 20 h.

Cells were pelleted by centrifugation at 3,500 g for 35 min at 4°C, resuspended in ∼100 mL of 50 mM KPi and 100 mM NaCl, pH 7.4. Once resuspended, the cell slurry was transferred to a stainless-steel beaker and 50 µM FAD was added and the suspension was allowed to equilibrate for ∼10 min on ice. A Branson 450 sonifier set to 40 W was used to lyse the cells (∼8 min on ice). Cellular debris was separated by centrifugation at 10,000 g for 1 h (4°C). The supernatant was loaded onto a Talon affinity column (10 × 1.25 cm) pre-equilibrated with 50 mM KPi, 100 mM NaCl, 10 mM imidazole, pH 7.4. The column was washed with 150 mL of the equilibration buffer and the protein was eluted using a 400 mL gradient from 10–300 mM imidazole in 50 mM KPi, 100 mM NaCl, pH 7.4. Eluent was collected as 5 mL fractions over the course of the elution and those with significant absorption at 280 nm were pooled and brought to room temperature. All subsequent manipulations were undertaken at this temperature. The pooled protein was concentrated using Amicon Ultra-15, 30 kDa centrifugal filters and FAD (∼500 µM) was added prior to being stored at −80°C. Prior to experimentation, the enzyme was thawed at room temperature and small amounts of precipitated enzyme were removed by centrifugation at 10,000 g for 10 min, and 30 kDa centrifugal filters were used to buffer exchange the supernatant enzyme into the desired reaction buffer (typically 50 mM KPi, pH 7.4). Additionally, a heat treatment (heating at 55°C for 5 min) was performed with variants that were sufficiently stable, to induce precipitation of apo enzyme (Smith et al., 2023). Two relatively less stable variants, U597S and Y296A, did not undergo this additional step. All SmTGR variants were quantified using the measured extinction coefficient of the FAD cofactor bound to the enzyme. Each variant’s extinction coefficient was determined according to published protocols (Smith et al., 2023). Approximately 10 µL of a concentrated enzyme sample was diluted into 50 mM NaPi, pH 7.4 and an absorption spectrum recorded. SDS (to 1.1%) was added to the same cuvette and the solution was heated to 55°C to denature the enzyme. Once fully denatured, a spectrum of the free FAD was acquired and corrected for dilution. Using the established extinction coefficient for free FAD (ε_450 nm_ = 11,300 M^−1^cm^−1^) the extinction coefficient of FAD bound to SmTGR was determined. Additionally, the ratio between 280 nm and 460 nm was recorded to confirm consistency amongst preparations. For variants that could withstand heat treatment, a 280/460 nm ratio of 9–10 was typical. However, the less stable and untreated U597S and Y296A variants had a ratio of 11–12, suggesting there is contaminating apo-enzyme in these samples.

### Expression and purification of SmTrx

SmTrx was expressed, purified, and quantified using methods previously established (Smith et al., 2023).

### Anaerobic techniques and transient-state conditions

Following published protocols (Moran, 2019), anaerobic enzyme samples were prepared in glass tonometers by alternating between cycles of vacuum and pure argon while attached to a Schlenk line. All transient-state experiments measure absorption changes and were performed using a HiTech stopped-flow spectrophotometer (TgK Scientific) at 20°C. Buffers and substrate solutions were made anaerobic by sparging with argon for 5 min. Prior to experimentation (at least 3 h), an anaerobic solution containing 1 mM glucose and 1 U/mL glucose oxidase was prepared in a tonometer, as described above, and placed onto the stopped-flow instrument to remove residual molecular oxygen. The addition of glucose and glucose oxidase were observed to destabilize SmTGR and therefore glucose oxidase scrubbing of reaction solutions was not performed, and the previously loaded scrubbing solution was displaced with anaerobic buffer prior to loading the enzyme onto the instrument. Unless otherwise stated, all experiments were performed in 50 mM KPi, pH 7.4.

For each variant the reductive half-reaction was observed using both photomultiplier (PMT) detection at individual wavelengths specific to the observation and using charge coupled device (CCD) detection for all wavelengths between 300 and 800 nm. To provide adequate temporal resolution to reveal the complexities of all events observed when monitoring SmTGR under transient-state conditions, stopped-flow data were collected for multiple timeframes. This data was then spliced together at the limit of the shorter acquisition to form a continuous trace. Single wavelength data were fit individually using linear combinations of exponential terms according to Equation 1, using KaleidaGraph software. The rate constants returned are analytical and reflect event time frames and thus delineation but not intrinsic rates of individual reaction steps. In this equation X is the wavelength of observation, A_n_ is the amplitude associated with phase n, *k*_*obsn*_ is the observed rate constant for phase n, t is time in s and C is the absorbance end point.

$$A_{Xnm}=\left(\sum_{n=1}^{x}{∆A}_{n}\left(e^{-k_{obsn}t}\right)\right)+C$$ (1)

Where feasible, single wavelength data for which NADPH was titrated were fit using numerical integration in KinTek explorer software to descriptive models derived from conclusions drawn from analytical fitting to both single and multi-wavelength datasets. Where possible, extinction coefficients for initial states and changes in extinction coefficient for known oxidation and reduction steps were used to validate the values returned by the software. Arrows shown on plots that depict titration series experiments indicate either increasing concentration or increasing pH.

**FIGURE 3:**
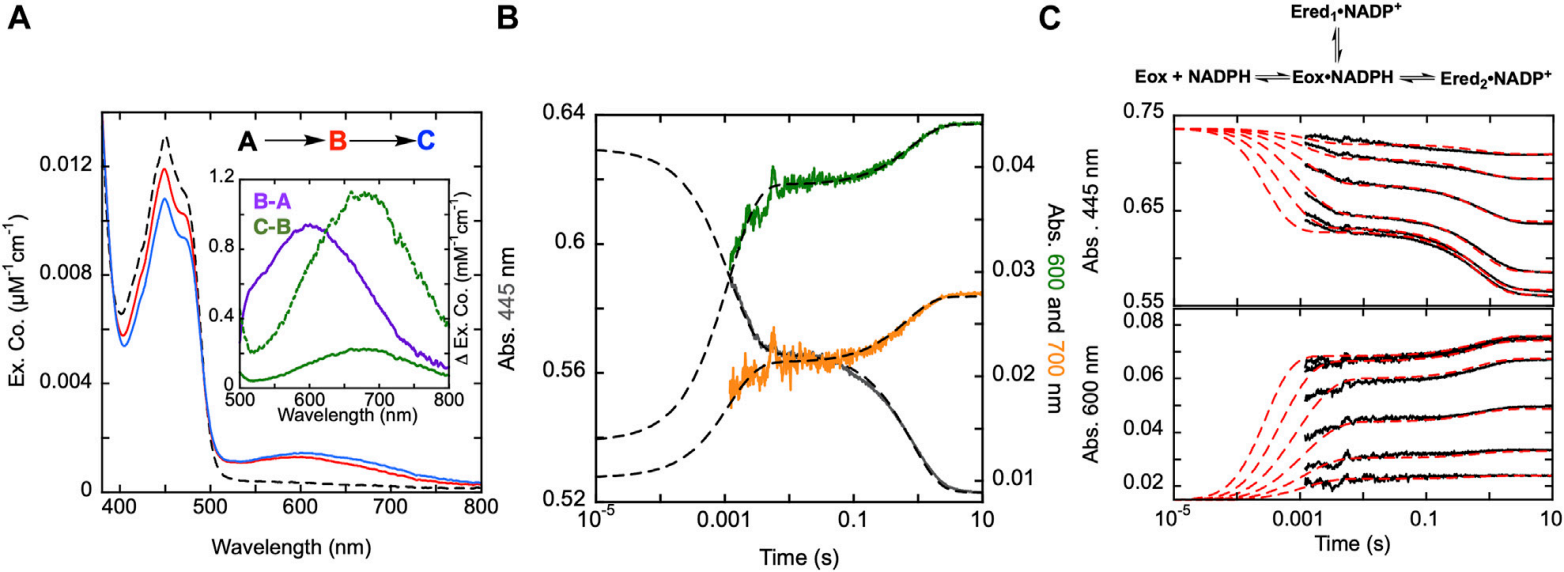
Reductive half-reaction of SmTGR C159A variant. **(A)**. SmTGR C159S (49 µM) was mixed with NADPH (256 µM) and the reaction was observed using CCD detection. The t_zero_ spectrum (dashed) was generated based on collected spectra of SmTGR and NADPH, and this was incorporated into the dataset. Two timeframes were collected (0.0016–1.6 s and 0.0016–16 s) and these were spliced together at 1.6 s. SVD was utilized to deconvolute the combined dataset by fitting to the two-step irreversible model shown. The inset depicts the extinction coefficient changes associated with each step of the model. The spectrum depicted with a dashed green line is the change associated with the second phase multiplied by five based on ∼20% fractional flavin reduction. **(B)**. SmTGR C159S (50 µM) was mixed with NADPH (48.3 µM) and the reaction was observed at 445 (grey), 600 (green), and 700 nm (orange). Two timeframes were collected (0.0012–0.2 s and 0.0012–10 s) and these were spliced together at 0.2 s. All traces were fit to a linear combination of two exponentials according to Eq. 1. All fits were extrapolated to their estimated t_zero_ absorbance based on the combined initial TGR and NADPH spectrum in order to estimate the amplitude change of the first phase that was largely complete within the deadtime of the instrument (1.2 ms). **(C)**. SmTGR C159S (58 µM) was mixed with NADPH (9.5, 19, 37.5, 75, 150, and 309 µM) using a stopped-flow spectrophotometer and the reaction was monitored at 445 nm (top) and 600 nm (bottom). Each reaction was collected for two timeframes (0.0012–0.2 and 0.0012–10 s) and the traces shown were spliced together at 0.2 s. The data from both wavelengths were fit simultaneously using numerical integration to the model depicted above. In this model the extinction coefficent change at 445 nm for reduction of the flavin was fixed to 9000 M^−1^cm^−1^.

Three dimensional CCD datasets were fit using singular value decomposition (SVD) to models that return spectra for intervening states using KinTek Explorer software (KinTeK Corp.). The models and initial estimates for rate constants were chosen based on the trends for amplitude and rates returned from single wavelength analysis. In each case the model used is shown with the data presented. The models chosen utilize SVD functionality in a similar manner to analytical fitting of single wavelength data to exponentials. Irreversible linear models constrain the software to return observed rate constants and yield spectra for delineated states rather than all ligand complexes and intermediate states that occur in the reaction. Prior to fitting, each CCD dataset incorporated a t_zero_ spectrum compiled from the absorbance contribution of the enzyme and NADPH measured for stock solutions in the same experiment. This spectrum was added to the dataset as a t = 0.00001 s spectrum. This spectrum is shown as a dashed line in Figures 3, 5–8.

### SmTGR C159S reductive half-reaction

To understand the role of the C159 residue in SmTGR’s electron distribution, the reductive half-reaction of the SmTGR C159S variant was performed. SmTGR C159S (49 µM) was mixed with NADPH (256 µM) and the reaction monitored using CCD detection. The C159S reductive half-reaction was also observed at individual wavelengths using PMT detection where C159S (50 µM) was mixed with NADPH (48.3 µM). The reduction of the flavin was observed at 445 nm, 600 nm and 700 nm Based on the t_zero_ spectrum, the starting absorbance for each wavelength was known. Despite missing a considerable portion of the first phase due to the limitations of mixing, extrapolating the exponential fits to t_zero_ absorbance for each wavelength allowed for the visualization of the estimated total amplitude changes encountered.

Additionally, a similar experiment was completed by combining C159S (58 µM) and a range of NADPH concentrations (9.5–309 µM) and monitoring the reaction at 445 and 600 nm to determine how the two phases observed titrate with increasing NADPH concentration. Due to the rate of the first observed event and the limitation imposed by mixing, all fits were extrapolated back to the artificially derived t_zero_ absorbance.

### NMR observation of hydride exchange in the NADPH•SmTGR C159S complex

Fractional reduction of the FAD is common for all forms of SmTGR including the U597C enzyme. This suggests that the flavin has low potential such that hydride exchange would take place between NADP and FAD. Nuclear magnetic resonance (NMR) was used to track the 4-position hydrogens on the nicotinamide of NADPH in the presence of SmTGR C159S. The C159S variant lacks the ability to distribute electrons to the 596–597 disulfide and therefore retains electrons within NADPH•FAD and NADP^+^•FADH_2_ complexes. The reaction was performed in D_2_O. The 4-position hydrogens of NADPH are not exchangeable with solvent deuterons, but once transferred to the N5 position of the isoalloxazine ring of the flavin as a hydride, exchange with solvent deuterium is possible. As such if reversible hydride exchange were occurring the NADPH would become stereospecifically enriched with deuterium at the nicotinamide 4-position. The reaction was performed under anaerobic conditions to avoid the loss of the electrons to dioxygen.

Initially control NMR spectra were collected for the reaction buffer (50 mM KPi, dissolved in 100% D_2_O pH 7.4), 1 mM NADP^+^, and 1 mM NADPH. Each observation was performed using a Bruker Avance 500 NMR instrument fitted with a cryoprobe. All spectra reported included 128 free induction decays (FIDs) and were acquired over a period of 9 min and 20 s at 25°C. Within reaction buffer a solution of 1 mM NADPH and 1 µM *Aspergillus niger* catalase was prepared. Although the reaction mixture was prepared under anaerobic conditions, catalase was added to ensure that if reduced SmTGR were to react with any available dioxygen, the resulting H_2_O_2_ would quickly be disproportionated and moreover, this activity further depletes oxygen with the sample. A spectrum of the NADPH/catalase solution was collected. This was repeated after 20 min to ensure the solution was unchanging devoid of SmTGR. The same NADPH/catalase solution was made anaerobic in a glass tonometer (as described above) and was placed in a Plas-Laboratories 830 series glovebox. Once the glovebox was sealed, it was made anaerobic by flushing with pure nitrogen gas for approximately 30 min. A Forensics Detectors oxygen meter was used to measure the fractional dioxygen level inside the glovebox, which was determined to be 0.1% prior to initiating the reaction. SmTGR C159S (10 µM) was mixed with the 1 mM NADPH/1 µM catalase solution inside the tonometer (14.3 µL of the enzyme solution was diluted into 1 mL of the NADPH/catalase solution). The tonometer was then opened inside the glove box and the solution was transfer to an NMR tube and capped before being removed from the glove box. Approximately 9 min passed before collection of the NMR spectrum was initiated, with the first spectrum being obtained ∼18 min post mixing. Although the reaction appeared to be complete after the first spectrum was collected, repeated spectra were collected to ensure the solution was unchanging.

### SmTGR U597S reductive half-reaction

To demonstrate the effect of mutation of the U597 residue, the reductive half-reaction of the SmTGR U597S variant was undertaken. SmTGR U597S (16 µM) was mixed NADPH (130 µM) and the absorption changes were monitored using CCD detection. Additionally, the reaction was monitored at specific individual wavelengths. U597S (16 µM) and NADPH (5.7–80 µM) were mixed and the reaction was observed at 460 and 540 nm using photomultiplier detection. The 460 nm measurement was utilized to report reduction of the flavin, while the 540 nm observation was made to track the formation of broad thiolate-FAD charge transfer absorption (Huang et al., 2011; Smith et al., 2023).

### SmTrx titration to SmTGR U597S

To demonstrate the function of the 596–597 disulfide the reductive half-reaction of SmTGR U597S variant in the presence of the oxidant substrate Trx was observed. SmTGR U597S (20 µM) was mixed with a solution of NADPH (18.5 µM) and either 0 µM SmTrx or 178 µM SmTrx. To avoid the adventitious reduction of SmTrx by NADPH, the stopped-flow instrument was used in double mixing mode and the SmTrx, NADPH, and SmTGR solutions were loaded into individual drive syringes. The SmTrx and NADPH solutions were mixed and aged for 0.1 s and were then mixed with SmTGR. The reaction was monitored at 460 and 540 nm using photomultiplier detection.

### SmTGR C31S reductive half-reaction

The 28–31 disulfide lies adjacent to a putative GSSG binding site and is thought to be the terminal enzyme disulfide that when reduced by interaction with the mobile 596–597 thiols, delivers electrons to GSSG. To show whether the kinetic events observed previously with the U597C enzyme report 28–31 disulfide reduction, the C31S variant (20 µM) was mixed with NADPH (4.25–34.5 µM) and the reaction was observed at 460 and 540 nm using PMT detection. To reveal spectra of reaction states, SmTGR C31S (30.7 µM) was mixed NADPH (30.27 µM) and the reaction monitored using CCD detection.

### SmTGR Y296A reductive half-reaction

Tyrosine 296 is proposed to gate access of the NADPH nicotinamide to the flavin N5 position (Figure 2). Observation of the Y296A variant reductive half-reaction was initiated by mixing SmTGR Y296A (21.5 µM) and NADPH (4.6–139.5 µM) and was monitored at 455 and 540 nm. The Y296A reductive half-reaction was also monitored using CCD detection. For this experiment SmTGR Y296A (20.2 µM) was mixed NADPH (16.5 µM).

### SmTGR H571A reductive half-reaction

Histidine 571 extends from the adjacent subunit of the dimer such that its epsilon nitrogen is proximal to the sulfur of C159 (3.7 Å) (Figure 2). The equivalent residue of thioredoxin reductase (H245) has been studied as a candidate active site base that deprotonates the structurally equivalent cysteine to C159 (C138) to promote reactivity of the thiol pair (Mulrooney and Williams, 1994). Initial characterization of SmTGR H571A involved observation of the reductive half-reaction. SmTGR H571A (22 µM) and NADPH (4.5–73.5 µM) were mixed and the reaction was observed at 460 and 540 nm. Data collected at 460 and 540 nm were extrapolated to the predicted t_zero_ absorbance to demonstrate the portion of the traces missed due to the limitations of mixing for instrumentation used. Additionally, SmTGR H571A (20.6 µM) was mixed with NADPH (17 µM) and the reaction was monitored using CCD detection.

### SmTGR H571A pH titration

To understand the influence proton concentration has on the rate of formation of the broad charge transfer, which has previously been assigned to the proximity of the C159 thiolate and FAD isoalloxazine (Angelucci et al., 2010; Smith et al., 2023), pH titrations of the U597C and the H571A variants SmTGR reductive half-reactions were performed. U597C SmTGR (19.8 µM) was mixed in a pH jump experiment with ∼20 µM NADPH in 6X MAT buffer (300 mM acetic acid, 300 mM MES, 600 mM Tris) titrated with 14.6 M H_3_PO_4_ to various pHs and the reaction was monitored at 540 nm. SmTGR is not stable in lower ionic strength buffers and was therefore prepared in 1X MAT (50 mM acetic acid, 50 mM MES, 100 mM Tris) buffer pH 7.4. For each NADPH solution prepared at a given pH, the stock solution was diluted into 6X MAT buffer (4 mL) and immediately sparged with argon gas for ∼5 min. The solution was mounted to the stopped-flow instrument and a spectrum recorded to establish the concentration of NADPH prior to mixing with SmTGR. This was particularly important to ensure the concentration of NADPH was known for acidic conditions where loss of NADPH due to cyclization is expected (Wu et al., 1986). Once mixed, both buffers were halved in concentration, however the 1X MAT buffer used to dilute the enzyme has influence on the final pH of the reaction solution. Therefore, for each pH both buffers were mixed in a 1:1 ratio and the pH of this solution was measured such that the final pH was known. The range of pHs measured along with the corresponding NADPH concentrations for the U597C SmTGR are: 5.35 (18.22 µM NADPH), 6.12 (20.26 µM NADPH), 7.33 (19.36 µM NADPH), and 9.31 (21.9 µM NADPH).

To directly compare to the U597C SmTGR pH titration, similar methods were used to analyze the SmTGR H571A variant. SmTGR H571A (19.95 µM) was mixed with ∼20 µM NADPH at various pHs and the reaction was monitored at 540 nm. The range of pHs measured along with the corresponding NADPH concentrations for the SmTGR H571A variant were: 4.73 (13.64 µM NADPH), 5.20 (17.48 µM NADPH), 5.65 (18.3 µM NADPH), 6.16 (19.55 µM NADPH), 6.85 (20.05 µM NADPH), 7.34 (20.2 µM NADPH), 7.95 (20.8 µM NADPH), 8.59 (23.8 µM NADPH), and 8.88 (21.0 µM NADPH). The first phase of the traces collected for the pH range from 5.20–8.88 were fit to single exponential according to Equation 1. The resulting *k*_obs_ values were plotted against pH and these data were fit to Equation 2. In this equation, X represents the value of the titratable phenomenon (in this case *k*_*1obs*_) and X_AH_ and X_A-_ represent the fully protonated and unprotonated arms of the titration respectively.

$$X=\frac{X_{AH}\left[H^{+}\right]+K_{a}X_{A-}}{\left[H^{+}\right]+K_{a}}$$ (2)

### Reduction of SmTGR H571A by NADPH under acidic conditions

The fully reduced SmTGR spectrum has proven difficult to obtain. Low reduction potential, non-native reductant molecules do not readily reduce SmTGR (data not shown). In addition, the enzyme did not tolerate photoreduction in the presence of EDTA (Massey et al., 1978; Massey and Hemmerich, 1978). Furthermore, when utilizing NADPH as the source of electrons the fully reduced state of the enzyme is not achieved. The pH titration demonstrates that when using the H571A variant the formation of the thiolate-FAD charge transfer can be diminished at more acidic pHs. Therefore, the SmTGR H571A variant was utilized to obtain the spectrum of fully reduced SmTGR. In an anaerobic cuvette, a spectrum of ∼11.5 µM H571A diluted into 1X MAT buffer, pH 5.0, was collected using a Shimadzu 2600 spectrophotometer. The enzyme was mixed with 1 mM NADPH (∼65.8 µL added, prepared in tris base). Repeated spectra were collected until the solution was virtually unchanging. Once the enzyme was fully reduced, the solution was then mixed with 6X MAT buffer, pH 10.28 (∼1 mL) to demonstrate the formation of the broad charge transfer absorption in more basic conditions. After this spectrum was collected, the anaerobic cuvette was opened, and the pH of the final solution was measured to be ∼9. All spectra were corrected for dilution.

## Results

### SmTGR C159S reductive half-reaction

Published X-ray crystal structures of SmTGR reveal what appears to be a chain of three cysteine pairs which could act as electron carriers to move electrons derived from NADPH to specific parts of the enzyme to reduce oxidant substrates (Figure 1; Figure 2) (Sandalova et al., 2001; Angelucci et al., 2010). Positionally, the first of these is the C154-C159 disulfide pair that is nearest to the *si*-face N5 position of the isoalloxazine ring of the FAD (Figure 2). Prior work suggests that the reduction of this disulfide and deprotonation of the most proximal thiol (C159) is required to enhance disulfide exchange reactivity toward the subsequent sulfide-selenide pair (C596-U597). The positionally equivalent residue from *E. coli* GR (C47) has been mutated and shown to be required for FAD-thiolate charge transfer formation (Rietveld et al., 1994). In SmTGR reduction of the 154–159 disulfide is also thought to induce the thiolate-FAD charge transfer absorption, which can be observed independent of the flavin transitions at 540 nm (Smith et al., 2023). Therefore, the SmTGR C159S variant was prepared to verify the source of this charge transfer absorption and to halt the ingress of electrons at the flavin cofactor. Using CCD detection, the reductive half-reaction of SmTGR C159S in the presence of excess NADPH was monitored (Figure 3A). The data collected were deconvoluted using SVD, fitting the data to the linear two-step irreversible model shown. Consistent with observations made at single wavelengths for this reaction (discussed below), this data fit to *k*_*1*_ of 730 ± 18 s^−1^, and *k*_*2*_ of 1.46 ± 0.01 s^−1^ revealing three spectra that represent the spectrophotometric changes associated with the C159S reductive half-reaction (Figure 3A). In Figure 3A inset the difference spectra calculated by subtracting each spectrum from the subsequent, reflect the spectral changes occurring in each phase. The data show that the C159S variant resolves an additional state that is not observed with the SmTGR U597C enzyme presumably as flavin reduction in this form of the enzyme is rapid and this absorption feature is overlapped and additive with the thiolate-FAD charge transfer (Smith et al., 2023). This added broad absorption band is centered around 600 nm and is largely formed within the deadtime of the instrument concomitant with partial (10%) flavin reduction. This feature has an extinction coefficient of ∼1000 M^−1^cm^−1^ and is assigned as charge transfer arising from the proximity of the NADPH dihydronicotinamide ring and the flavin isoalloxazine. This event decreases the net absorption at 340 nm (not shown) and at 445 nm, consistent with NADPH oxidation and flavin reduction. A second flavin reduction phase is then observed to occur with similar amplitude. We offer no explanation for the apparent biphasic reduction of the flavin with this variant. Based on the estimated extinction coefficient change used to quantify flavin reduction (∼9000 M^-1^cm^-1^ at 450 nm) (Crozier-Reabe and Moran, 2012), reduction of an additional 10% of the sample is observed in the second phase. Importantly, the thiolate-FAD charge transfer arising after flavin reduction in the U597C enzyme is not observed, implicating C159 as directly contributing to this absorption. The initial charge transfer absorption appears to be maintained in the second phase. The reason for this is that only 20% of the enzyme reduces at equilibrium such that the majority of the flavin remains in the oxidized state in the presence of unreacted and/or reinstated NADPH (that for this experiment was added in large excess). Additionally, a charge transfer feature with longer wavelength absorption characteristics (maximum absorbance around 685 nm) is observed to accumulate concomitant with the second fractional flavin reduction. That this absorption is only observed as more reduced SmTGR C159S accumulates indicates its origin is the proximity of FADH_2_ and NADP^+^. With only 20% reduction it is apparent that the NADPH and flavin are in redox equilibrium and that the flavin has the more negative potential. When corrected for the fraction reduced, the extinction coefficient of this second charge transfer band is estimated to be ∼1100 M^−1^cm^−1^ at 680 nm (dashed spectrum in Figure 3A inset).

The reductive half-reaction of the SmTGR C159S variant was also monitored at single wavelengths (Figure 3B). SmTGR C159S was mixed with approximately equimolar NADPH and the reaction was monitored at 445, 600, and 700 nm to track each of the unique signals observed in this reaction. However, as the absorption of both charge transfers overlap, (Figure 3A), 600 and 700 nm were respectively selected as the approximate isosbestic point and a wavelength of significant extinction coefficient difference. Each wavelength was fit to a linear combination of two exponentials returning rate constants of, *k*_*1*_ = 887–779 s^−1^, and *k*_*2*_ = 1.31–1.23 s^−1^. As a visual cue for the reader, where each trace begins is defined by the t_zero_ spectrum. Collectively these data are consistent with two unique charge transfer states that form at different rates. As suggested from the CCD data, the charge transfer with absorbance centering around 600 nm forms with NADPH complexation in the first phase and is sustained throughout the rest of the observation, and the second charge transfer absorption (absorbance centering around 685 nm) fractionally accumulating within the second phase as the enzyme becomes further reduced.

The extent to which observed rate constants titrate with NADPH concentration was established by mixing SmTGR C159S with NADPH and the reaction was monitored at 445 and 600 nm (Figure 3C). The data for both wavelengths was fit to a branching three step reversible model using numerical integration. Though largely obscured by the instrument deadtime, the first phase appears to titrate in both rate and amplitude with increasing NADPH concentration with the rate coming to a limit. Thus, NADPH binding equilibrium is attained within the deadtime but influences the observed rate(s) of the first phase which fits to report a K_NADPH_ of 7 ± 0.9 µM. The subsequent event is similar in rate for all NADPH concentrations, but increases in amplitude with increasing NADPH concentration, again suggesting that NADPH and FAD are in redox equilibrium such that more enzyme is reduced as NADPH concentration increases from mass action. The fit returned values for the initial hydride transfer of *k*_*2*_ = 2,530 ± 1,230 s^−1^ and k_-2_ = 12,374 ± 2,486 s^−1^ followed by a slower net reduction of the flavin with rates of *k*_*3*_ = 0.33 ± 0.05 s^−1^ and k_-3_ = 1.3 ± 0.1 s^−1^.

### Evidence for hydride exchange equilibrium between FAD of the C159S variant and NADPH

When observing the reductive half-reaction of the U597C SmTGR, partial flavin reduction and incomplete NADPH oxidation has consistently been observed (Smith et al., 2023). Additionally, in the absence of oxidants and with limiting concentrations of NADPH, rather than pass all of the available electrons to the disulfides, the FAD will remain partially reduced suggesting a redox equilibrium between NADPH and the disulfides in which the flavin acts a conduit in both directions. We have proposed that in addition to electrons from NADPH being distributed between the flavin and the cysteine pairs, electrons also return to NADP^+^, regenerating NADPH and limiting flavin reduction. To demonstrate a rapid hydride exchange equilibrium between NADPH and FAD, the SmTGR C159S variant was employed as it is unable to distribute electrons beyond the flavin.

**FIGURE 4:**
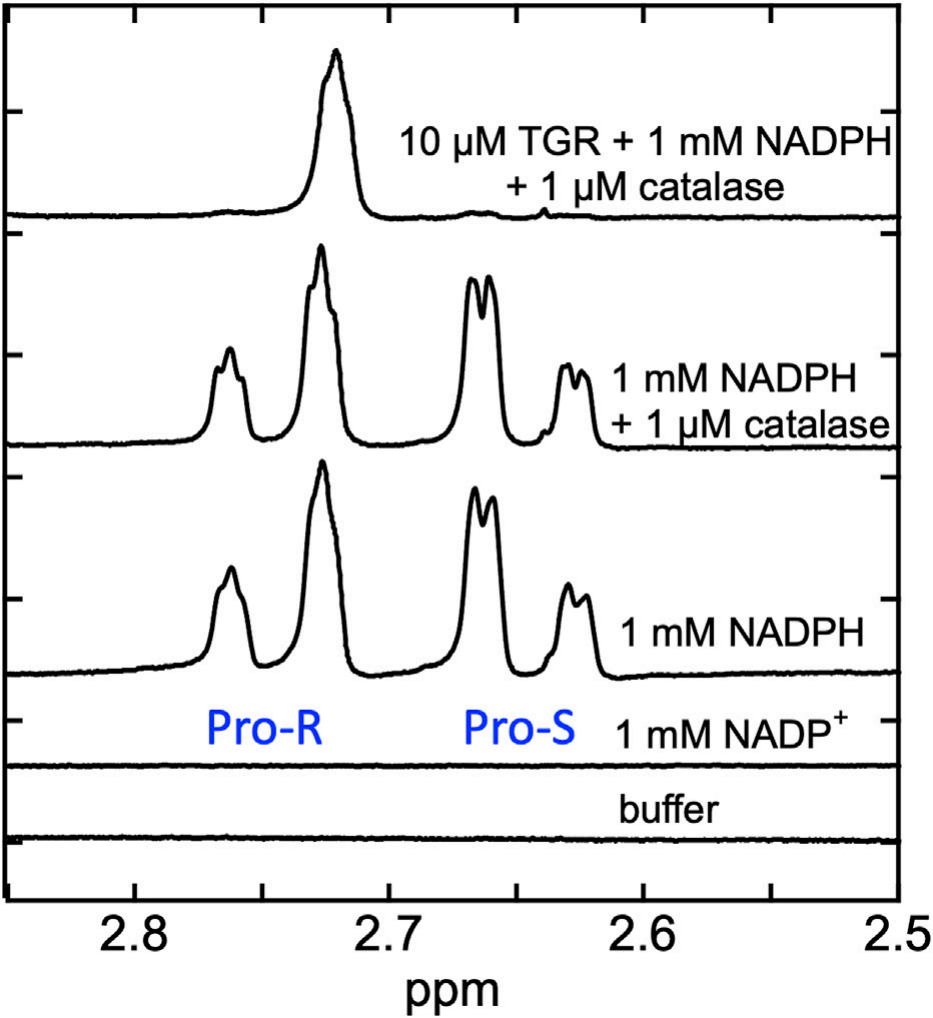
Demonstration of equilibrium hydride exchange between NADPH and FAD in the SmTGR C159S variant. Proton NMR was used to measure the rate of exchange of electrons between NADPH and FAD of SmTGR. Standard spectra of 50 mM KPi in 100% D_2_O pH 7.4 buffer, 1 mM NADP^+^ prepared in buffer, 1 mM NADPH prepared in buffer, and 1 mM NADPH and 1 µM *Aspergillus niger c*atalase prepared in buffer are shown for reference. The same solution of 1 mM NADPH and 1 µM catalase was made anaerobic and was placed inside a glove box. Nitrogen was flushed through the system until 0.1% O_2_ was measured. SmTGR C159S (10 µM) was mixed with the NADPH/catalase solution and was transferred into an NMR tube and capped. A spectrum was obtained 18 min post mixing (total time required to mix and remove sample from glove box, prepare instrument and collect spectrum). Repeated spectra were collected to be sure this solution was unchanging. All spectra measured were collected by obtaining 128 FIDS.

Proton NMR spectra were recorded for SmTGR C159S in the presence of NADPH in buffered D_2_O. The rationale was that hydride transfer to the flavin N5 delivers the proton from the ostensibly non-exchangeable aliphatic 4-position of the NADPH dihydronicotinamide to the amine N5 position of the flavin isoalloxazine that is exchangeable with solvent. Exchange with solvent at the N5 position of the flavin hydroquinone during reversible hydride transfer should then deplete protons from one face of the NADPH dihydronicotinamide such that it becomes stereo-specifically labeled with deuterium. In Figure 4 we demonstrate that the control spectrum collected for NADPH has two pairs of signals (between 2.85–2.5 ppm) from the two C4 hydrogens of the dihydronicotinamide. After the addition of SmTGR C159S (∼10 µM), the first spectrum collected (∼18 min post mixing) reveals that all NADPH (1 mM) has been converted to [4S-^2^H] NADPD. This establishes that equilibrium hydride transfer between FAD and NADPH is occurring consistent with an apparent low reduction potential for the active site flavin. The orientation of NADPH within the crystal structure of the SmTGR (U597C)•NADPH complex (Figure 2), along with a previously performed kinetic isotope effect with Pro-S NADPD (Smith et al., 2023), establishes that the hydride transferred to the FAD of SmTGR from NADPH is from the Pro-S position and permits assignment of the observed NMR signals.

### Characterizing the function of C597 residue

**FIGURE 5:**
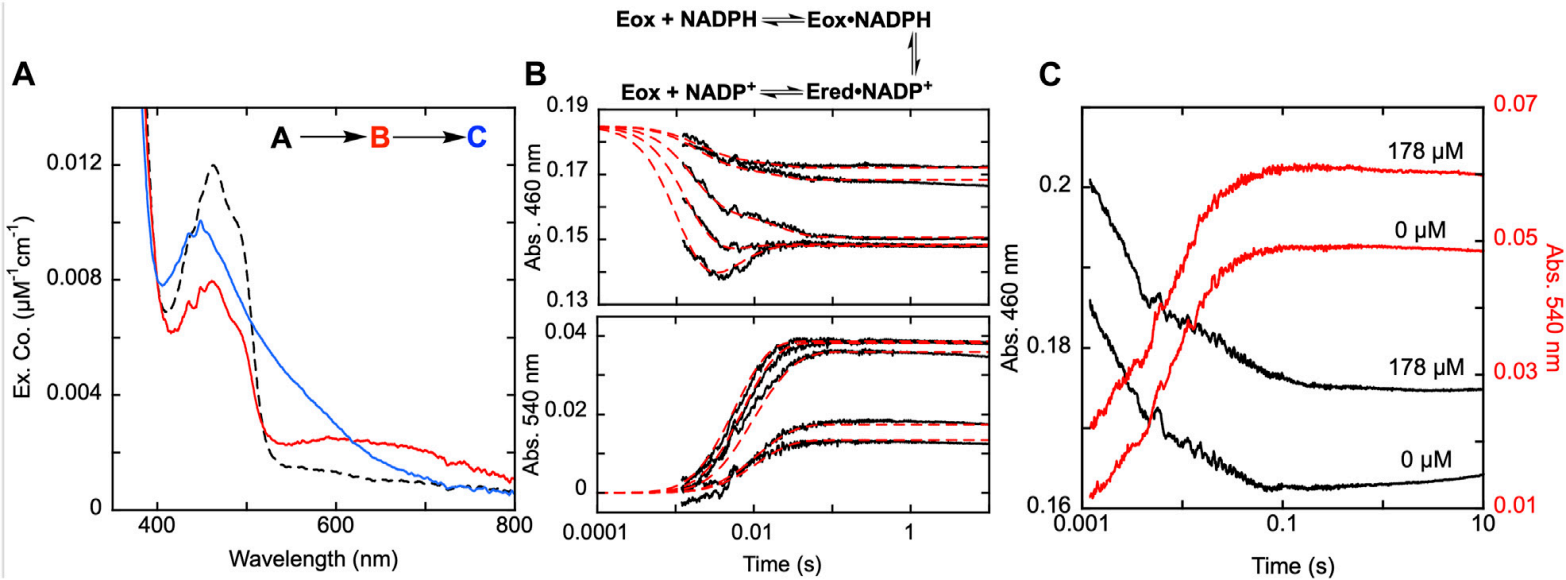
Reductive half-reaction of SmTGR U597S variant. **(A)**. SmTGR U597S (16 µM) was mixed with NADPH (130 µM) and the reaction was observed using a CCD detector. The t_zero_ spectrum was generated based on the combined initial spectra of SmTGR and NADPH and was incorporated into the dataset. Two timeframes were collected (0.0016–1.6 s and 0.0016–16 s) and these were spliced together at 1.6 s. The dataset was deconvolute by utilizing SVD by fitting to the two-step irreversible model shown. **(B)**. SmTGR U597S (16 µM) was mixed with NADPH (5.7, 7.5, 20, 40, 80 µM) and the reaction was monitored at 460 nm (top) and 540 nm (bottom). Two timeframes were collected (0.0012–0.2 and 0.0012–10 s) and the traces shown were spliced together at 0.2 s to provide adequate time resolution for all phases. The data from both wavelengths were fit simultaneously using numerical integration to the model depicted above. In this model the extinction coefficent change at 445 nm for reduction of the flavin was fixed to 8000 M^−1^cm^−1^. **(C)**. SmTGR U597S (20 µM) was mixed with NADPH (18.5 µM) solution that contained 0 µM or 178 µM SmTrx. The reaction was observed at 460 and 540 nm. Two timeframes were collected (0.0012–0.2 and 0.0012–10 s) and the traces shown were spliced together at 0.2 s. The traces were separated for clarity.

SmTGR has two oxidant binding sites. Comparison of the TrxR•Trx complex structure with that of SmTGR allows for the distance between these sites to be estimated at ∼45 Å (Figure 1). The C596-U597 cysteine pair is thought to bridge these oxidant binding sites acting as a tethered mobile mediator between the C154-C159 disulfide, the disulfide of the SmTrx substrate (C34-C37) and the C28-C31 disulfide adjacent to the GSSG binding site. In order to formally demonstrate the role of the C596-U597 disulfide, the 597 residue was mutated to a serine (U597S) and we characterized the effect of this change on the reductive half-reaction. SmTGR U597S was mixed with NADPH and the reaction was observed using a CCD detector. The data set was deconvoluted using SVD, fitting to a two-step irreversible model shown in Figure 5A. The fit reported rate constants of 930 ± 50 s^−1^ and 164 ± 5 s^−1^. The first event consists of FAD reduction and the formation of a charge transfer absorption with a maximum at 640 nm. Due to the limiting fractional extent of flavin reduction (40%) this absorption cannot be definitively assigned to either NADPH-FAD or NADP^+^-FADH_2_ charge transfer. Moreover, the absorption maximum is roughly centered between the spectra observed for these states as they were assigned in Figure 3A suggesting the spectrum captured is a mixture of these states. The second phase indicates loss of the 640 nm charge transfer absorption concomitant with accumulation of the broadly absorbing thiolate-FAD charge transfer. Overall, when comparing this data to that collected for U597C SmTGR (Smith et al., 2023), the initial steps of the reductive half-reaction are similar. However, the U597S variant is unable to move electrons beyond the C154-C159 pair and therefore the thiolate-FAD charge transfer is sustained, confirming the proposed role of the C596-U597 disulfide.

In Figure 5B, SmTGR U597S was mixed with a range of NADPH concentrations and the reaction was monitored at 460 nm and 540 nm using photomultiplier detection. Both datasets were fit simultaneously to the model shown, that includes NADPH binding followed by two reversible steps, the latter of which included NADP^+^ release. Similar to other variants, the 460 nm data reveal an initial phase which appears to titrate with increasing NADPH concentration. Unlike the SmTGR U597C variant for which the flavin is observed to begin reoxidizing at ∼0.1 s in the presence of limiting NADPH concentration (Smith et al., 2023), this variant maintains the partially reduced flavin and thiolate-FAD charge transfer state. The data is adequately described by this simple mechanism that amounts to a truncated version of what is observed for the U597C enzyme. The fit of the data reports a dissociation constant for NADPH of 20 ± 1 µM followed by hydride exchange between the flavin and NADPH with rate constants in both directions close to 1,000 s^−1^ to predict an observational rate constant of ∼2000 s^−1^, broadly similar the conclusions drawn from the CCD data (Figure 5A). The ensuing phase is assigned tentatively as NADP^+^ dissociation as formation of the thiolate-FAD absorption has been demonstrated to be contingent on dissociation of this product for the U597C enzyme.

Although it was previously established that SmTrx will alter the observed reoxidation of the U597C SmTGR variant, impacting the first observed flavin reoxidation phase rate and ultimately eliminating the second reoxidation phase at higher Trx concentrations (Smith et al., 2023), it was not hitherto established whether the C154-C159 or the C596-U597 disulfides act to donate electrons to this oxidant. Figure 5C displays the reductive half-reaction of SmTGR U597S variant in the presence and absence of SmTrx. This data clearly demonstrates that there is no change to the observed kinetics when excess SmTrx is combined with SmTGR U597S in the presence of limiting NADPH, establishing that the C596-U597 disulfide is required for SmTrx reduction.

### The SmTGR C31S reductive half-reaction

**FIGURE 6:**
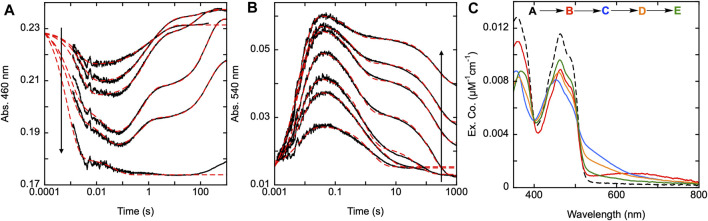
Reductive half-reaction of SmTGR C31S variant. SmTGR C31S (20 µM) was mixed with NADPH (4.25–34.5 µM) and the reaction was monitored at 460 nm **(A)** and 540 nm **(B)**. Data collected at 540 nm were all brought to the same starting point for visual clarity. The lowest concentration (4.25 µM) was collected for two timeframes (0.0012–0.2 s and 0.0012–200 s) while all other concentrations were collected for three timeframes (0.0012–0.2 s, 0.0012–100 s, and 0.0012–1,000 s). All the traces shown were spliced together at 0.2 s and 100 s, when applicable. All data were fit to a combination of exponentials according to Eq. 1. For the 460 nm data, the 4.25 and 34.5 µM NADPH concentrations were fit to three and two exponentials, respectively, while the other concentrations were fit to four exponentials. All fits were extrapolated back to the t_zero_ absorbance at 460 nm. The 540 nm traces were fit to a linear combination of two (4.25 µM) or three (8.25–34.5 µM) exponentials. **(C)**. SmTGR C31S (30.7 µM) was mixed with NADPH (30.27 µM) and the reaction was observed using a CCD detector. The t_zero_ spectrum was generated based on collected spectra of SmTGR and NADPH alone, and this was incorporated into the dataset. Three timeframes were collected (0.0016–1.6 s, 0.0016–160 s, and 0.0016–1,600 s) and these were spliced together at 1.6 s and 160 s. SVD was utilized to deconvolute the combined dataset by fitting to the four-step irreversible model shown.

As previously stated, the C28-C31 pair is thought to be the disulfide responsible for the reduction of GSSG, as GSH has been observed to bind adjacent to this disulfide (Angelucci et al., 2010). Transient state kinetic studies of the SmTGR U597C variant revealed a turnover number for SmTGR with GSSG as the oxidant substrate to be slower than any of the processes observed under net single turnover conditions (Smith et al., 2023). Additionally, when titrating GSSG to the enzyme in the presence of limiting NADPH (relative to SmTGR concentration) and observing the resulting net single turnover reaction, there appears to be little change to the observed kinetics. This indicates that although the C28-C31 pair may participate in the reduction of GSSG, its involvement is not evident in the transient state kinetic observations we report. In Figure 6, we observed the reductive half-reaction of the SmTGR C31S variant to discern if the kinetics were impacted by this mutation. SmTGR C31S was mixed with NADPH and the reaction was monitored at 460 and 540 nm. The 460 nm data were fit to a linear combination of exponentials according to Equation 1. Complexity in this data precludes numerical integration fitting to a comprehensive model for the entire data set. This complexity has been shown to arise from interdimer redox activity that occurs with reduction of the mobile C596-C597 disulfide. This phenomenon is additive with internal redox events and collectively is too multifarious to meaningfully account for in numerical integration fitting. As expected, this data was very similar to that collected for the U597C SmTGR variant with each delineated event occurring on similar time frames (Smith et al., 2023). The range of *k*_*obs*_ values for this data set include: *k*_*1*_ of 560–1,30 s^−1^, *k*_*2*_ of 125–159 s^−1^, a *k*_*3*_ of 0.3–3.1 s^−1^, and a *k*_*4*_ of 0.005–0.02 s^−1^.

Additionally, SmTGR C31S was mixed with limiting NADPH and the reaction was monitored using CCD detection. The dataset was deconvoluted by fitting to the model shown in Figure 6C which is the same linear irreversible model used in prior work to fit similar CCD datasets collected for U597C SmTGR (Smith et al., 2023). The C31S SVD deconvoluted spectra reveal an initial phase in which flavin reduction and long-wavelength charge transfer (absorption centering around 660 nm) accumulate with a rate constant of 840 ± 40 s^−1^. This was then followed by a loss of the long wavelength charge transfer absorption and formation of the more intense shorter wavelength thiolate-FAD charge transfer transitions with a rate constant of 129 ± 2.1 s^−1^. Two reoxidation phases follow with rate constants of 2.5 ± 0.1 s^−1^ and 0.006 ± 0.001 s^−1^, with the final spectrum collected demonstrating incomplete flavin reoxidation. Based on the estimated extinction coefficient change of 9000 M^−1^cm^−1^ at 450 nm for flavin reduction, the most reduced state obtained appears to be ∼34% reduced and at equilibrium ∼16%. As such, the C31S variant is a facsimile for observations made for the U597C SmTGR (observed to be ∼35% reduced at its most reduced state and ∼14% reduced at redox equilibrium) (Smith et al., 2023). This shows that eliminating the C28-C31 disulfides ability to receive electrons does not alter the observed kinetics of the reaction, thus defining the last reoxidation phase observed in the U597C net single turnover reaction to be associated with electron distribution through the C596-U597 C-terminus and not the interaction with the C28-C31 disulfide.

### The reductive half-reaction of the SmTGR Y296A variant

In 2018, Silvestri et al. identified a residue within SmTGR adjacent to the NADPH binding site that was critical for the mode of action of specific inhibitors (Silvestri et al., 2018). When examining the crystal structure of the SmTGR•NADPH complex (PDB 2X99) one can see that the Y296 residue is found in a conformation stacked with the NADPH nicotinamide that is roughly parallel to the flavin. Comparing this to SmTGR structures lacking NADPH (PDB 2X8C), the phenol of Y296 is oriented approximately perpendicular to the isoalloxazine re-face, aligned with the central N1-N5 axis, and overlapping with the NADPH binding site (Figure 2). This is structural evidence that movement of Y296 is associated with binding of NADPH and suggests that this residue may be acting to block the active site FAD from non-specific reduction and/or reoxidation and thus confines the redox activity of reduced SmTGR. Structural evidence for the movement of a phenol group in a tyrosine residue, oriented roughly perpendicular to the FAD with the binding of NADPH has also been reported for GR (Karplus and Schulz, 1987) and similar positioning in TrxR has also been implied (Silvestri et al., 2018). Krauth-Siegel et al., studied the Y197S variant of GR and concluded the diminished accumulation of FAD-NADPH charge transfer and the overall decrease in the rate constants observed for reduction were evidence of Y197 directly participating in the flavin reduction event (Krauth-Siegel et al., 1998).

**FIGURE 7:**
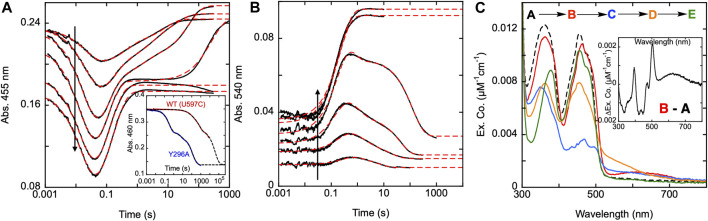
Reductive half-reaction of SmTGR Y296A variant. SmTGR Y296A (21.5 µM) was mixed with NADPH (4.6–139.5 µM) and the reaction was monitored at 455 nm **(A)** and 540 nm **(B)**. The vertical positioning of the 455 nm traces was adjusted for clarity. All concentrations were collected for three timeframes (0.0012–0.2 s, 0.0012–10 s, and 0.0012–300 s) while the 36.5 µM concentration was additionally collected from 0.0012–2000 s. All traces were spliced together at 0.2, 10, and 300 s, when applicable. Data were fit to a linear combination of exponentials according to Eq. 1. Within the 455 nm dataset the lowest NADPH concentration (4.6 µM) was fit to three exponentials, 9.2, 18.5, and 36.5 µM NADPH concentrations were fit to four exponentials, and the two highest concentrations (71 and 139.5 µM) were fit to two exponentials. The 540 nm data were fit to three exponentials, except for the two highest (71 and 139.5 µM NADPH) which were fit to one exponential. The inset in A compares the rate of dithionite reduction for the U597C and Y296A variants. For both, enzyme (29 µM) was mixed with 1.4 mM dithionite under anearobic conditions. The data were fit to three exponentials according to Eq. 1. **(C)**. SmTGR Y296A (20.2 µM) was mixed with NADPH (16.5 µM) and the reaction was observed using a CCD detector. The t_zero_ spectrum was generated based on the combined initial spectra of SmTGR and NADPH alone, and this was incorporated into the dataset (dashed line, species A). Three timeframes were collected (0.0016–1.6 s, 0.0016–16 s, and 0.0016–316 s) and these were spliced together at 1.6 and 16 s. SVD was performed to deconvolute the dataset by fitting to the four-step irreversible model shown. The inset depicts the difference of species A and **(B)**.

To determine the functionality of the Y296 residue in SmTGR, a study of the reductive half-reaction of the Y296A variant was performed. SmTGR Y296A was mixed with a range of NADPH concentrations and the reaction was monitored at 455 and 540 nm (Figures 7A,B). As was the case for C31S, these data were fit analytically to exponentials as a descriptive approach to define only observed rate constants for the apparent phases. For analytical fitting of the 455 nm data according to Equation 1, the lowest NADPH concentration was fit to a linear combination of three exponentials, while the traces ranging from 9.2–36.5 µM NADPH were fit to four exponentials. As both 71 and 139 µM NADPH concentrations were in excess relative to SmTGR, these samples were over reduced, show no evidence of flavin reoxidation, and could be fit to two exponentials. The first phase observed appears to titrate both in amplitude and rate with increasing NADPH concentration. The Y296A variant has a considerably slower rate for flavin reduction than observed with the U597C, and the exponential fits to this event could not be extrapolated to the same t_zero_ absorbance value suggesting that a rapid-equilibrium binding event precedes it. This reaction was also monitored at 540 nm to track the formation and decay of the thiolate-FAD charge transfer absorption (Figure 7B). The 540 nm data are also consistent with initial rapid equilibrium binding in that the initial value at the time equivalent to the instrument deadtime increases and appears to saturate with increasing NADPH concentration. Except for the two highest concentrations (71 and 139.5 µM NADPH), which were fit to one exponential, 540 nm traces were fit to a linear combination of three exponentials according to Equation 1. The range of *k*_*obs*_ values for individual phases at both wavelengths are: *k*_*1*_ of 45–61 s^−1^, *k*_*2*_ of 2.0–10 s^−1^, a *k*_*3*_ of 0.6–3.5 s^−1^, and a *k*_*4*_ of 0.003–0.16 s^−1^.

To establish if the Y296 residue serves to shield the FAD from non-specific reduction, both the U597C and Y296A variants were mixed with millimolar concentrations of dithionite under anaerobic conditions (Figure 7A inset). The data obtained show the Y296A variant reducing more rapidly than the U597C enzyme, indicating that the phenol serves to hinder non-specific reduction. The data are multiphasic presumably due to electrons flowing toward the flavin both from dithionite and reduced portions of the disulfide exchange relay that acquire electrons in the presence of dithionite. Though it is not possible to know which phase corresponds to direct reduction of the FAD by dithionite the rate of reduction differs by at least 175-fold when comparing the initial rate observed for Y296A with the most rapid rate observed for the U597C enzyme.

The fundamental conclusion is that the rate of the flavin reduction by NADPH is approximately 30 to 45-fold slower than that observed with U597C SmTGR (or the C31S variant), implicating Y296 as directly influencing the rate of hydride transfer. In addition, for this variant, the thiolate-FAD charge transfer maximally accumulates at ∼0.8 s, a time frame that is roughly 10-fold slower than that of the U597C variant. This phase does correspond well to the second phase (first oxidation phase) observed in the 455 nm traces, confirming that reoxidation of the FAD and formation of the thiolate-FAD charge transfer absorption occur concomitantly, establishing that deprotonation of C159 remains rapid. As it was previously reported, NADP^+^ must dissociate from the active site prior to deprotonation of the C159 residue that forms the thiolate-FAD charge transfer complex, implicating roles for Y296 in the efficiency of reduction and in the dissociation of NADP^+^.

Additionally, using CCD detection, the reaction of SmTGR Y296A and limiting NADPH was monitored. This data set was deconvoluted using SVD by fitting to the linear model indicated, that includes four irreversible steps (Figure 7C). The binding event proposed above was included as a single step (*k*_*1*_) with a fixed rate constant in excess of 10,000 s^−1^. Given that no time dependent change describing this process is evident in the data, the value was chosen solely to ensure the event is complete within dead-time, consistent with the observation. The fit returned rate constants of *k*_*2*_ of 47.7 ± 1.5 s^−1^, a *k*_*3*_ of 9.1 ± 0.3 s^−1^, and a *k*_*4*_ of 0.05 ± 0.01 s^−1^. Figure 7C depicts the deconvoluted spectra, which represent the additive spectrophotometric characteristics of all states present at the junctions between phases. Subtracting the first deconvoluted spectrum from the second returns a difference spectrum shown in Figure 7C inset. Based on our assignment, this spectrum represents the binding difference spectrum for formation of the SmTGR Y296A•NADPH complex and includes NADPH-FAD charge transfer absorption centered around 625 nm. By permitting capture of evidence for a state with flavin spectral perturbation and long-wavelength charge transfer prior to NADPH oxidation and flavin reduction, the Y296A variant confirms what was proposed for the C159S above (Figure 3A). This variant has exhibited the most completely reduced intermediate spectrum of SmTGR yet observed, with approximately 90% of the FAD being reduced (based on an assumed extinction coefficient change of 9000 M^-1^cm^−1^), with limiting NADPH concentration. This strongly suggests that Y296 is involved in the binding and release of NADPH/NADP^+^. This event is then followed by the formation of the thiolate-FAD charge transfer and ultimately incomplete reoxidation of the flavin.

### The reductive half-reaction of the SmTGR H571A variant

As previously stated, kinetic studies of U597C SmTGR variant and the data shown above for the C159S variant implicate that an intense, broad charge transfer, which we have typically observed at 540 nm, is the result of the proximity of the C159 thiolate and oxidized FAD isoalloxazine (Smith et al., 2023). This thiolate arises from the reduction of the C154-C159 disulfide and the subsequent deprotonation of the 159 sulfhydryl. The available X-ray crystal structures implicate histidine 571 (that arises from the opposing subunit) as being responsible for this deprotonation, as it is the only residue that is proximal to the C154-C159 pair that has the capacity to act as a general base (Figure 2). The equivalent residue in *E. coli* TrxR (H245) and human GR (H467) have been implicated in this role without being associated with the formation of FAD-thiolate charge transfer (Mulrooney and Williams, 1994; Krauth-Siegel et al., 1998). However, Rietveld et al., showed that mutation of H439 in GR from *E. coli* suppressed formation of FAD-thiolate charge transfer (Rietveld et al., 1994). In lipoamide dehydrogenase H450 is positioned similarly to accept a proton from the nascent C48 residue that is released by reduction (Mattevi et al., 1991).

**FIGURE 8:**
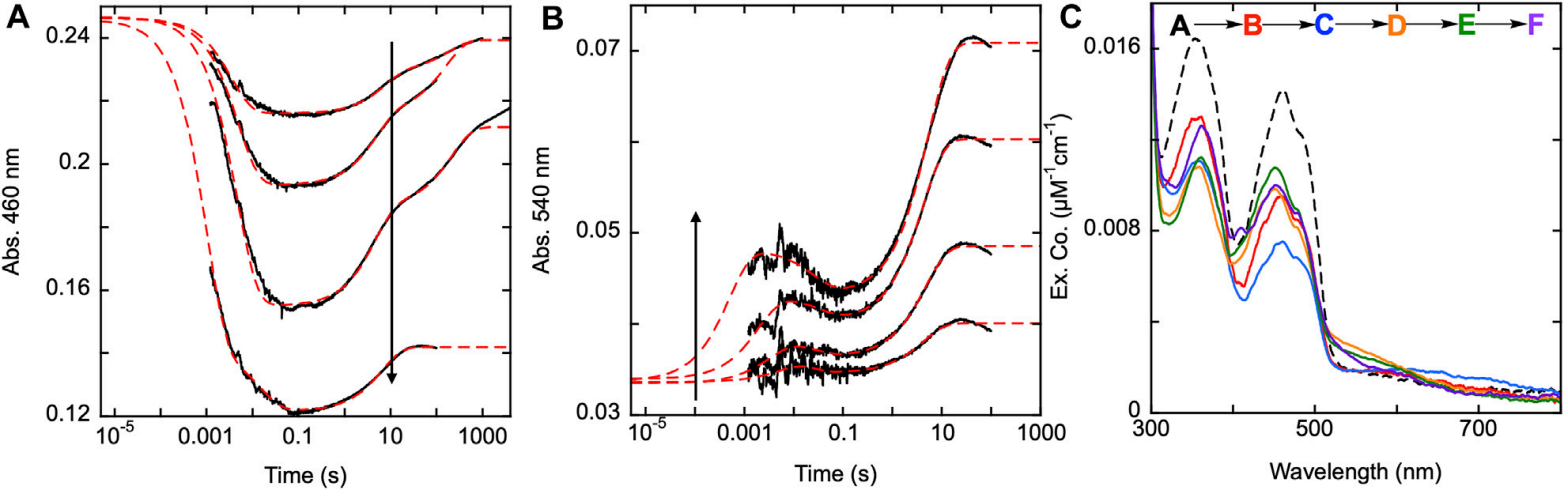
Reductive half-reaction of SmTGR H571A variant. SmTGR H571A (22 µM) was mixed with NADPH (4.5–73.5 µM) and the reaction was monitored at 460 nm **(A)** and 540 nm **(B)**. All concentrations were collected for three timeframes (0.0012–0.2 s, 0.0012–10 s, and 0.0012–100 s), while the 4.5 µM concentration was additionally collected from 0.0012–1,000 s and the 19 µM concentration was collected from 0.0012–4,000 s. All traces were spliced together at 0.2, 10, and 100 s, when applicable. All data were fit to a linear combination three exponentials according to Equation 1, with the fits extrapolated to the t_zero_ absorbance at each wavelength. **(C)**. SmTGR H571A (20.6 µM) was mixed with NADPH (17 µM) and the reaction was observed using CCD detection. The t_zero_ spectrum was generated based on the combined initial spectra of SmTGR and NADPH alone, and this was incorporated into the dataset (dashed line, species A). Three timeframes were collected (0.0016–1.6 s, 0.0016–160 s and 0.0016–7,900 s) and these were spliced together at 1.6 s and 160 s. SVD was used to deconvolute the combined dataset by fitting to the five-step irreversible model shown.

To confirm this apparent role, SmTGR H571A was mixed with a range of NADPH concentrations and was monitored at 460 and 540 nm using PMT detection (Figures 8A,B). For this variant, absorption changes associated with flavin reoxidation are exceptionally slow. In most instances the data were collected on a 100 s time frame, however reoxidation can be observed over 10,000 s. For a specific instance (19 µM NADPH) data were collected for 4,000 s and were revealed to not be at equilibrium (Figure 8A). Expanded time frames to 8,000 s were used for CCD detection discussed below. The 100 s data collected at 460 nm, were fit analytically to a linear combination of three exponentials with extrapolation to the computed t_zero_ absorbance. The range of *k*_*obs*_ values were: *k*_*1*_ of 203–282 s^−1^, *k*_*2*_ of 0.13–0.22 s^−1^, and a *k*_*3*_ of 0.005–0.007 s^−1^. These ranges exclude the rate constant values determined for the highest concentration of NADPH (73.5 µM) as over-reduction changes the observed kinetics. In addition, note that the *k*_*3*_ value is derived from the slowest event observed at this wavelength for longer time frame traces and was not captured in the 540 nm data described below that are all limited to 100 s. For this reason, the observed *k*_*2*_ value at 450 nm corresponds to the observed *k*_*3*_ at the longer wavelength. The highest NADPH concentration used in this reaction was ∼3.5-fold higher than the enzyme concentration and the flavin reduction phase does not appear to approach a limit in terms of rate or amplitude. Figure 8B displays the data collected at 540 nm with all traces also being fit analytically to a linear combination of three exponentials. All exponential fits were extrapolated to the computed t_zero_ absorbance at 540 nm. The range of *k*_*obs*_ values for this data were: *k*_*1*_ of 105–2000 s^−1^, *k*_*2*_ of 27–91 s^−1^, and a *k*_*3*_ of 0.19–0.24 s^−1^. The first species observed at 540 nm is presumably charge transfer absorption arising from localization of the NADPH dihydronicotinamide adjacent to the FAD isoalloxazine. This feature then decays and the species that forms is then logically assumed to be a state in which the C154-C159 disulfide is reduced and C159 is not yet deprotonated. On a much longer timeframe a third phase is observed which corresponds to accumulation of thiolate-FAD charge transfer transitions (observed rate constant of ∼0.2 s^-1^). This indicates that it is probable the H571 residue is necessary for the rapid formation of the thiolate-FAD charge transfer by deprotonation of C159. In the absence of the 571 imidazole, C159 is deprotonated but at a ∼17-fold slower rate, indicating that the 159–571 position(s) is relatively insulated from interaction with solvent.

The H571A reductive half-reaction was also monitored using CCD detection. SmTGR H571A was mixed with a limiting concentration of NADPH and the reaction was observed for 8,000 s and so incorporates an additional phase that is not evident in the single wavelength data (Figure 8A). SVD was utilized to deconvolute the spectra by fitting the data to the linear five step irreversible model indicated (Figure 8C). These data do not capture a spectrum which resembles formation of the SmTGR•NADPH complex. Instead, the first event reflects only reduction of the flavin, NADPH oxidation, and the accumulation of a small charge transfer absorption with absorbance centering around 650 nm. This spectrum indicates roughly 60% of the flavin is reduced. The second event reports FAD oxidation and accumulation of the thiolate-FAD charge transfer absorption albeit with considerably less intensity than observed for U597C, U597S, C31S, or the Y296A variants (Figure 5A; Figure 6C; Figure 7C). In the next state the flavin has partially reoxidized and the thiolate-FAD charge transfer absorption is diminished. This suggests a redistribution of electrons away from the FAD and thiolate such that some fraction of the C154-159 disulfide is reformed. This is then followed by an apparent flavin reduction phase that includes increases around 360 nm. This slow phase is indicative of equilibrium overshoot, where electrons initially flow into the enzyme and then fractionally return to NADPH at equilibrium. The data fits to rate constants of: *k*_*1*_ of 204 ± 2 s^−1^, *k*_*2*_ of 0.22 ± 0.01 s^−1^, a *k*_*3*_ of 0.005 ± 0.001 s^−1^, and a *k*_*4*_ of 0.00016 ± 0.0001 s^−1^. However, this sequence reflects the five-component limitation built into the analysis software and excludes the 27–91 s^−1^ steps observed at shorter time frames. To resolve this issue a 1,000 s dataset was fit to five components and the spectra shown reflect these five states plus one additional final state deconvoluted from the 8,000 s dataset. Together these data agree with the observations made when monitoring this reaction at single wavelength and confirms that the thiolate-FAD charge transfer accumulates with an observed rate that is ∼20-fold slower and to lower extents when the enzyme is lacking the imidazole of the H571 residue.

### Effect of pH on the transient-state observations of the SmTGR H571A variant

**FIGURE 9:**
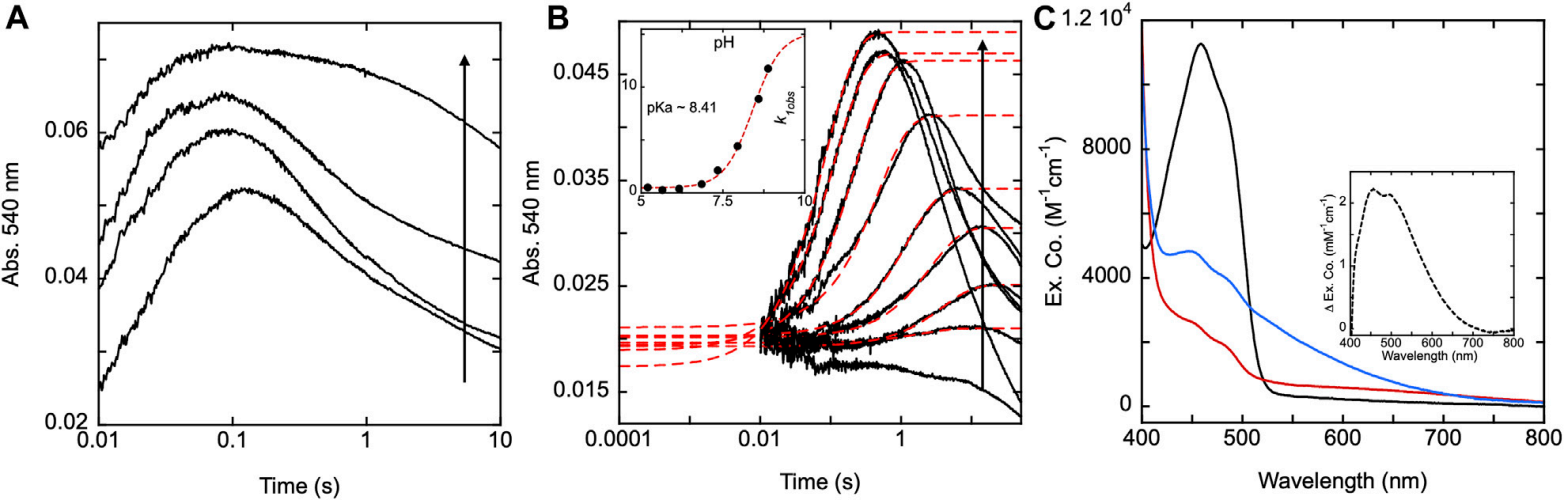
SmTGR U597C and H571A pH titrations. **(A)** SmTGR U597C (19.8 µM) prepared in 1X MAT buffer (0.5X final) pH 7.4 was mixed with 6X MAT buffer (3X final) at various pHs in the presences of ∼20 µM NADPH and the reaction was monitored at 540 nm. To determine the pH for which each reaction was observed, both buffers were mixed in a one-to-one ratio and the pH was measured, with the titration displayed ranging from pH 5.35–9.31. All data were collected for three timeframes (0.0012–0.2 s, 0.0012–1 s, and 0.0012–10 s) and were spliced together at 0.2 and 1 s **(B)** SmTGR H571A (19.95 µM) prepared in 1X MAT buffer (0.5X final on stopped-flow) pH 7.4 was mixed with 6X MAT buffer (3X final) at various pHs in the presences of ∼20 µM NADPH and the reaction was monitored at 540 nm. To determine the pH for which each reaction was observed, both buffers were mixed in a one-to-one ratio and the pH was measured, with the titration displayed ranging from 4.73–8.88. All reactions were collected for three timeframes (0.0012–1 s, 0.0012–10 s, and 0.0012–100 s) and the data was spliced together at 1 and 10 s. The first phase of the traces obtained for pH 5.20–8.88 were fit to a single exponential according to Eq. 1. The *k*_*1obs*_ values were then plotted against pH and this data was fit to Eq. 2 (inset). **(C)** SmTGR H571A (∼11.5 µM) was diluted into 1X MAT buffer pH 5.04, prepared under anaerobic conditions, and a spectrum was collected on a Shimadzu UV-Vis 2,600 (black). While maintaining the anaerobic conditions, ∼1 mM NADPH prepared in low strength Tris base was mixed with the enzyme to over reduced the it and a spectrum was collected (red). The enzyme was then mixed with ∼1 mL 6X MAT buffer pH 10.28 and a spectrum was collected (blue). The final pH of the solution was measured with pH paper to be roughly pH 8–9. All spectra were corrected for dilution. The inset depicts the difference between the blue and red spectra.

To show the effect pH has on the SmTGR H571A reductive half-reaction kinetics, the enzyme was mixed using a pH-jump method with limiting NADPH at various pH values. The reaction was monitored at 540 nm to specifically observe the impact that pH had on the rate of thiolate-FAD charge transfer formation for this variant. A control reductive half-reaction with the U597C SmTGR enzyme showed no clear evidence that the observed kinetics for formation of the thiolate-FAD charge transfer absorption titrate significantly with pH (5.35–9.31) indicating that activation of the C159 residue is not influenced by the pH of the solvent (Figure 9A). Under the conditions indicated, SmTGR H571A was mixed with NADPH and the final pHs of the reaction ranged from 4.73–8.88 (Figure 9B). This data demonstrates that when the H571 residue is mutated to an alanine, the 159 sulfhydryl is now subject to proton equilibrium with the solvent, albeit at a slow rate. The slow accumulation of the FAD-thiolate charge transfer indicates that the si face of the isoalloxazine is largely secluded from solvent such that equilibrium deprotonation of C159 is achieved over relatively long timeframes. Moreover, the rate of formation of the thiolate-FAD charge transfer titrates in a manner consistent with base activation of a cysteine residue in that the rate increases with pH and exhibits a dependence with an apparent pKa of 8.4 (Figure 9B inset).

The near complete suppression of thiolate-FAD charge transfer at low pH for the H571A variant provided an opportunity to determine the isolated absorption spectrum of this feature. With exception of the C159S variant, attempts to fully reduce any form of SmTGR with excess NADPH or other reductants are complicated by the intense broad absorption of the thiolate-FAD charge transfer that is additive with residual flavin absorption. SmTGR H571A was prepared under anaerobic conditions in 1X MAT buffer at pH 5.04 and a spectrum was recorded (Figure 9C). With the introduction of excess NADPH the enzyme rapidly reduced without evidence of thiolate-FAD charge transfer. The resulting change in extinction coefficient at 450 nm was approximately 8000 M^−1^cm^−1^, similar to the estimated extinction coefficient of 9000 M^−1^cm^−1^ we have used to estimate fractional reduction throughout. Subsequently, the enzyme solution was mixed with 6X MAT buffer pH 10.28 (volume ratio 1:2) to increase the pH of the solution and induce the formation of the thiolate-FAD charge transfer. The difference spectrum was calculated (Figure 9C inset) by subtracting the initial low pH reduced spectrum from the subsequent high pH reduced spectrum, yielding the net changes associated with the deprotonation of the thiol under basic conditions. This spectrum shows that the thiolate FAD has a maximum extinction coefficient of ∼2000 M^−1^ cm^−1^ at ∼450 nm and so skews estimates of fractional reduction of the SmTGR flavin.

## Discussion

Platyhelminthes of the genus *Schistosoma* infect humans and other mammals leading to the debilitating and frequently lethal disease schistosomiasis (van der Werf et al., 2003a; WHO, 2023). This syndromatic condition can be effectively treated with the ion channel activator praziquantel. However, the multiple large doses required for cure and limited resources for distribution in what are often remote areas leads to incomplete or suppressive treatment regimes in place of curative. Moreover, *Schistosoma* parasites are endemic to many tropical and subtropical regions and reinfection is common (van der Werf et al., 2003b; Steinmann et al., 2006; Utzinger et al., 2009). There is evidence of resistance to praziquantel (Utzinger et al., 2009; Wang et al., 2012) and given that it is the only approved and effective treatment for schistosomiasis, an imperative exists to develop new treatments. Recently the enzyme thioredoxin/glutathione reductase (TGR) has been identified as a potential target as schistosomes have been shown to be highly reliant on this enzyme to combat the host immune response (Colley and Secor, 2014). TGR combines the catalytic functions of two enzymes, thioredoxin reductase (TrxR) and glutathione reductase (GR), utilizing an FAD and three disulfide pairs to transport electrons to thioredoxin (Trx) or glutathione (GSSG). Within this study, several variant enzymes of *Schistosoma mansoni* TGR (SmTGR) were studied to define the role of purported participants in the electron distribution pathway.

**FIGURE 10:**
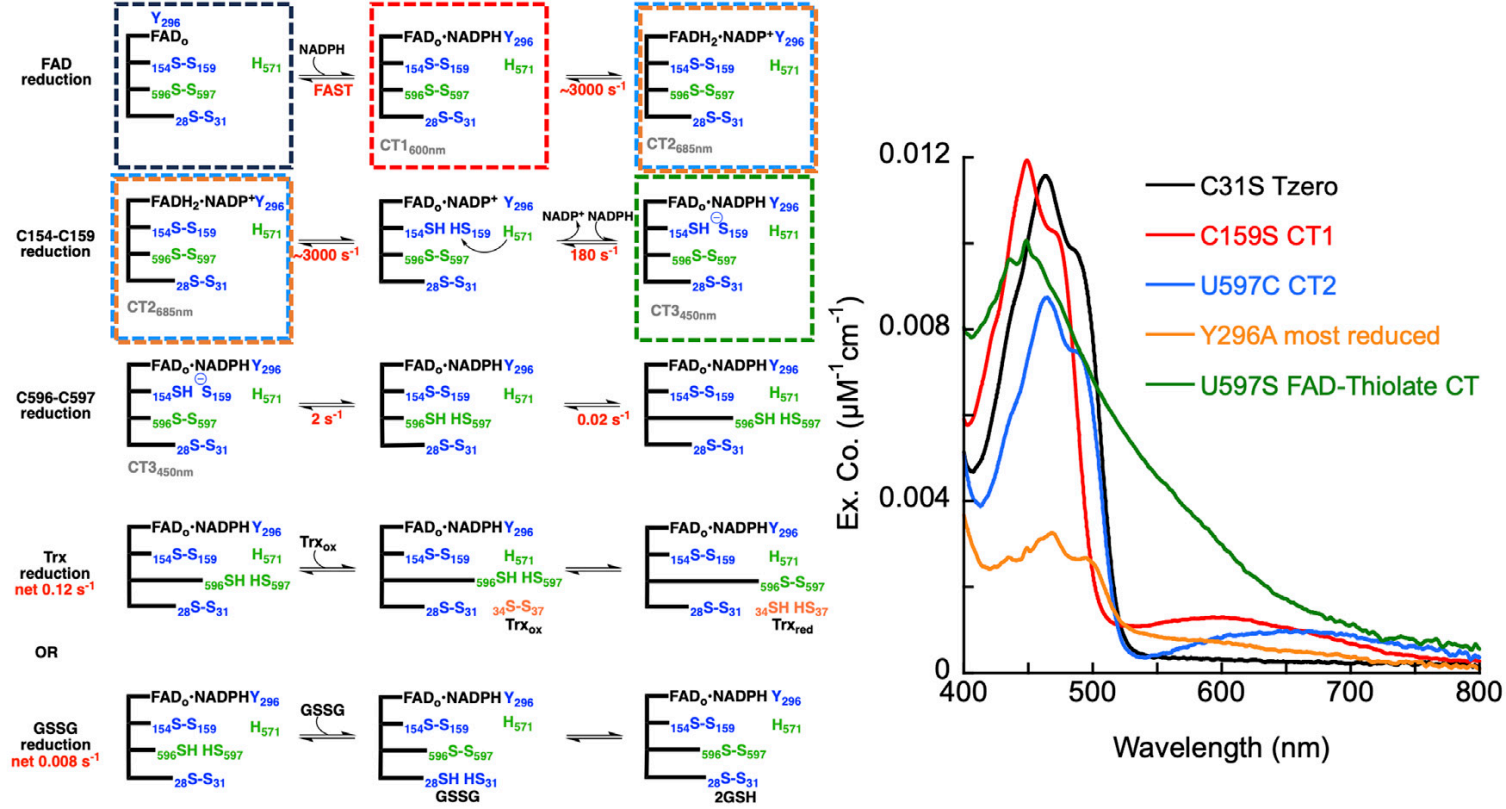
**(A)**. Proposed catalytic sequences of SmTGR. All rate constants indicated are observed and were determined from the U597C variant (Smith et al., 2023). No specific chemistry is assigned to the 0.02 s^−1^ step associated with C596-C597 reduction. Scaffolds represent participating substrates, residues and cofactors. The length of individual prongs qualitatively represents the requirement for movement. Residues rendered in blue are from subunit 1 and those in green are from subunit 2. **(B)**. Composite Sequence of Absorption Spectra. Spectra that exemplify discrete states taken from kinetic analysis of individual variants are shown as a composite sequence that approximates the events observed in the reductive half reaction of SmTGR including only events through deprotonation of C159 resulting in the formation of FAD-thiolate charge transfer. The order of events follows the legend from top to bottom. At wavelengths below 400 nm these spectra include contributions from added NADPH which varies with each experiment and the extent of reduction observed.

Figure 10 depicts a segmented catalytic mechanism of SmTGR. All rate constants indicated are observed rates determined from a recent transient state kinetic study of the U597C enzyme (Smith et al., 2023). In this scheme, blue colored residues (Y296, C154, C159, C28, and C31) represent those from subunit 1 of the dimer, while the green residues (C596, C597, and H571) are provided by subunit 2. Although these rate constants do not report intrinsic values, they are used to distinguish individual catalytic events and will be referenced when discussing the effect of the variants on specific catalytic steps. Multiphasic fractional flavin reduction and reoxidation observed during reductive half-reactions suggests all redox participants are constantly exchanging electrons and protons forming transient quasi-equilibrium states as opposed to discrete successive states on a reaction pathway. As such many more additional states than are depicted in Figure 10 are possible. This scheme is therefore a simplification depicting the passage of two electrons based on observations where the NADPH concentration was limiting with respect to the concentration of SmTGR. The data presented here suggest that TGR is a conduit for electrons in the forward and reverse directions and therefore attuned to the redox state of the cell. Data that support this will be discussed chronologically with respect to the sequence shown in Figure 10. For the most part, mechanistic assignments are based on interpretation of recurring identifiable absorption signals, particularly charge transfer absorption, in combination with analytical fitting, numerical integration fitting and spectral deconvolution.

The first state depicted in Figure 10 is that of the fully oxidized enzyme with all cysteine pairs as disulfides and the Y296 residue in a blocking conformation. The cosubstrate, NADPH, is shown to bind and displace the phenol of Y296. This conformational change was observed in comparison of crystal structures of partially reduced SmTGR (PDB 2X8C) and the SmTGR•NADP(H) complex (PDB 2X99) and results in a stacked arrangement for the FAD, NADPH and Y296 phenol (Figure 2) (Angelucci et al., 2010; Silvestri et al., 2018). When examining Figure 7C it was determined that the first species to be captured with SVD deconvolution of the SmTGR Y296A variant reductive half-reaction was that of the SmTGR•NADPH complex (inset). Therefore, the mutation of the Y296 residue slows flavin reduction and, in terms of rate, delineates this from the binding of NADPH. The first spectrum captured reflects spectral perturbation consistent with the binding of NADPH adjacent to the flavin without evidence of NADPH oxidation or flavin reduction. Additionally, a charge transfer absorption band centered around 625 nm is observed to form with binding. At long wavelengths the deconvoluted spectrum closely resembles the character of the first species captured for SmTGR C159S that also showed charge transfer absorption centered around 600 nm (Figure 3A). Again, this data is consistent with changes associated with the binding of NADPH proximal to the FAD cofactor and therefore this initial charge transfer absorption has been assigned to occur with the formation of the SmTGR•NADPH complex for the oxidized state of the flavin. Although the rate constants associated with the binding of NADPH are not defined in this analysis, estimates of the K_NADPH_ could be obtained when the NADPH concentration series was fit for both the C159S and U597S variants (Figure 3C; Figure 5B, respectively). The K_NADPH_ values determined from these fits were 7 ± 0.1 µM (C159S data) and 20 ± 1 µM (U597S data).

After the association of NADPH, the flavin readily accepts electrons. With the exception of C159S and Y296A, this event is very rapid with estimated observed rate constants of 1000 s s^−1^, and for this reason a large portion of the flavin reduction phase is generally obscured in the deadtime of the stopped-flow instrument. However, when analyzing the reductive half-reaction of the C159S variant we can clearly observe the flavin reduction event (Figure 3). Concomitant with flavin reduction, a second charge transfer absorption is observed to form with its absorbance centering around 685 nm (Figure 3A). This charge transfer absorption is present as the first observed spectrum in the U597C variant reductive half reaction (Figure 10) and has previously been assigned to occur with stacking of the NADP^+^ nicotinamide and FAD isoalloxazine hydroquinone. However, despite being estimated to have similar extinction coefficient compared to the first charge transfer absorption (Figure 3A inset), the C159S variant is observed to only fractionally reduce to ∼20% and the NADPH-FAD charge transfer absorption dominates the observation of initial events in the reductive half-reaction as a result of residual NADPH.

As stated previously, the U597C SmTGR enzyme and all other variants studied reduce to different extents during the initial flavin reduction phase (U597C and C31S reduce to ∼35%, C159S reduces to ∼10%, U597S reduces to ∼40%, Y296A reduces to ∼90%, and the H571A reduces to ∼60%). This suggests that the C159, Y296 and H571 residues influence the initial redox equilibrium between the flavin and the NADPH. Figure 4 confirms that there is indeed a hydride transfer equilibrium between the NADPH and the FAD cofactor in that complete exchange of the NADPH dihydronicotinamide Pro-S hydrogen is observed in buffered deuterium oxide in the presence of catalytic amounts of SmTGR C159S. This hints that the redox potential of the flavin is likely similar to that of NADPH and the variants modulate the balance of forward or reverse hydride transfer rates resulting in varied extents of flavin reduction. The Y296A variant, in particular, has tipped the balance in the forward direction such that the flavin is observed to ostensibly completely reduce (Figure 7C). The positioning of the 296 phenol suggests that in the absence of NADPH it orients to block access to the isoalloxazine N5 and prevent non-specific reduction or reoxidation of the flavin, kinetically restricting promiscuous/futile electron transfer out of the TGR population such that even low reduction potential non-native reductants fail to reduce the FAD significantly on timeframes relative to catalysis. The rapid rate of reduction by dithionite observed for the Y296A variant supports this role (Figure 7A inset).

Thus, following flavin reduction, electrons either flow backwards, regenerating NADPH, or will move further into the enzyme reducing the disulfide pair most proximal to the N5 position of the isoalloxazine ring of the FAD, C154-C159 (Figure 2). A prior model used to fit the U597C data (Smith et al., 2023), suggested that this disulfide reduction occurs on a similar time frame as flavin reduction and is therefore estimated to also have a rate constant on the 1000 s s^−1^ timescale. It has been previously demonstrated that, once this reduction occurs, NADP^+^ must dissociate to allow for the subsequent deprotonation of the C159 thiol to generate the thiolate-FAD charge transfer absorption (Smith et al., 2023). When analyzing the reductive half-reaction of the Y296A variant, the formation of this charge transfer absorption is slowed ∼10-fold (Figure 7). This suggests that not only is the Y296 residue influencing the binding of NADPH and subsequent flavin reduction, but it is likely also impacting the rate of dissociation of NADP^+^. Additionally, the H571 residue was implicated as being responsible for the deprotonation of the C159 thiol and a pH titration experiment completed with the H571A variant indicates that this thiol has a pka of ∼8.4 (Figure 8; Figure 9B). This data, along with the lack of formation of the thiolate-FAD charge transfer absorption when monitoring the C159S variant reductive half-reaction, confirms the residue responsible for this observed transition. When the imidazole moiety is absent a noticeable titration in both amplitude and rate of formation of the thiolate•FAD complex is observed. However, when performing a similar pH titration with the U597C variant minimal changes in the formation of the thiolate-FAD charge transfer are observed (Figure 9A) suggesting both that the 571 histidine residue is the acting general base and the C159 residue is highly shielded from solvent. An estimated spectrum of the thiolate-FAD charge transfer absorption was obtained by over reducing the H571A variant enzyme under acidic conditions and then ramping to higher pHs (Figure 9C inset), revealing an extinction coefficient of approximately 2000 M^−1^ cm^−1^ at 450 nm (referenced throughout as the 540 nm charge transfer, where it was conveniently observed independent of flavin absorption transitions).

In Figure 10 the next phase of catalysis is reduction of the C596-C597 disulfide by disulfide exchange. To further evaluate this process within the electron distribution chain of SmTGR, the U597S variant was studied. In Figure 5A the reductive half-reaction of the U597S variant demonstrates that the initial phase captured appears to be flavin reduction, concomitant with the formation of a long wavelength charge transfer absorption. This is then followed by the formation of the broad thiolate-FAD charge transfer absorption. However, as this variant enzyme is lacking the C596-C597 disulfide the thiolate-FAD charge transfer absorption is sustained, confirming that this disulfide is next in the sequence to receive electrons. This in turn implies that the first of the two phases for which the thiolate-FAD charge transfer diminishes for the U597C enzyme, with an approximate rate constant of 2 s^−1^, occurs as a result of the C154 C159 thiol/thiolate pair reducing the C596-C597 disulfide. Previously, the next phase of flavin reoxidation/loss of thiolate-FAD charge transfer absorption was assigned to the reduction of the distant C28-C31 disulfide which is thought to be responsible for the reduction of GSSG. However, when comparing the reductive half-reaction of the C31S variant (Figure 6) to that of the U597C enzyme it is immediately apparent that, although the reduction of this disulfide is proposed to be necessary for the reduction of GSSG, this process is not captured in the transient state kinetic data presented. This is consistent with the previously reported titration of GSSG during the reductive half-reaction of the U597C enzyme for which minimal kinetic changes were detected (Smith et al., 2023), suggesting that all phases observed in the transient state occur prior, are not contingent on, and are more rapid than the reduction of the C28-C31 pair or GSSG. Therefore, as Figure 10 reflects, this second reoxidation phase (with an estimated rate constant of ∼0.02 s^−1^) has been tentatively assigned to a conformational change with the movement of the reduced C596-C597 disulfide. From here the C596 C597 thiol pair can either reduce the C34-C37 disulfide of SmTrx or reduce the C28-C31 disulfide pair within the GSSG domain. In the prior study, a titration of the oxidant substrate, SmTrx, was performed (Smith et al., 2023). This data demonstrated that Trx would perturb only the first flavin reoxidation phase, but it was not known definitively whether the disulfides of Trx interact directly with the C154-C159 pair or with the C596-C597 pair. However, Figure 5C indicates that the reduction of the C596-C597 disulfide is required for the reduction of Trx. When titrating SmTrx to the U597C enzyme we observe a slowing in rate of the first reoxidation event, which approaches the rate of turnover of the enzyme with Trx as the oxidant (from ∼2 s^−1^ to ∼0.12 s^−1^), and a complete loss of the second slower reoxidation event. This suggests that when Trx is available, all electrons will move into Trx, effectively bypassing the more elaborate conformational change required to reduce the distant C28-C31 disulfide. Interestingly, although this is seemingly faster than the second reoxidation event it is still ∼16-fold slower than the observed initial reoxidation phase in the absence of oxidants. Therefore, while SmTGR’s interaction with Trx reduces the overall net reoxidation rate of TGR this cannot be assigned to a specific observed process. Similarly, while the overall turnover of SmTGR with GSSG as an oxidant substrate occurs on a slower timeframe (∼0.008 s^−1^) than that of the second reoxidation event of SmTGR in the absence of oxidants (∼0.02 s^−1^), this rate cannot be directly assigned to any process required for the reduction of GSSG.

## Conclusive remarks

The data presented suggest that TGR has a distributive mechanism for the movement of reducing equivalents and that all redox active entities are in constant exchange such that electrons flow forward and backward resulting in staged reduction and reoxidation events that involves multiple redox active groups fractionally at any point in time. From a transient state analysis standpoint this, added to inter-dimer redox exchange, creates complexity that cannot be reasonably accounted for. As such we have chosen to describe SmTGR largely analytically based on observed rate constants for reductive and oxidative phases induced by the addition of limiting amounts of NADPH. These conditions cannot be said to provide or approximate requirements for first order kinetics and so do not yield intrinsic rates for any step observed. However, using recognizable spectrophotometric features that are indicative of the catalytic sequence, most particularly three charge transfer absorption transitions, permits assignment of the order of specific common catalytic events that in turn has indicated function for four of the five variants studied.

With the exception of C31S, the SmTGR variants studied each reveal the role of the parent residue. Along with C154, C159 is required to move electrons off the flavin and onto the C596-U597 disulfide and C159 is the source of the thiolate-FAD charge transfer absorption. The C159S variant limits electron distribution to the flavin revealing evidence for the association of NADPH and in addition yielded definitive evidence for reversible hydride exchange between the flavin and NADPH. The C596-U597 pair is required to reduce Trx as the thiolate-FAD charge transfer is sustained in the U597S variant even in the presence of excess Trx. Y296 is directly involved in the stacking of the NADP nicotinamide with the flavin isoalloxazine and flavin reduction is slowed when this residue is replaced with an alanine. H571 is required for rapid deprotonation of C159 and the H571A variant showed that the C159 residue becomes titratable with pH now modulating formation of the thiolate-FAD charge transfer band.

## Data Availability

The original contributions presented in the study are included in the article/supplementary material, further inquiries can be directed to the corresponding author.
