# Corrigendum: Assigning function to active site residues of *Schistosoma mansoni* thioredoxin/glutathione reductase from analysis of transient state reductive half-reactions with variant forms of the enzyme

**DOI:** 10.3389/fmolb.2024.1455473

**Published:** 2024-07-23

**Authors:** Madison M. Smith, Graham R. Moran

**Affiliations:** Department of Chemistry and Biochemistry, Loyola University Chicago, Chicago, IL, United States

**Keywords:** flavoprotein, disulfide, reductase, transient state, kinetics, variant

In the published article, there was an error in the **Funding** statement. A **Funding** statement was not included. However, the researchers who carried out the work were supported by federal grant funds. The correct **Funding** statement appears below.

